# Premetazoan genome evolution and the regulation of cell differentiation in the choanoflagellate *Salpingoeca rosetta*

**DOI:** 10.1186/gb-2013-14-2-r15

**Published:** 2013-02-18

**Authors:** Stephen R Fairclough, Zehua Chen, Eric Kramer, Qiandong Zeng, Sarah Young, Hugh M Robertson, Emina Begovic, Daniel J Richter, Carsten Russ, M Jody Westbrook, Gerard Manning, B Franz Lang, Brian Haas, Chad Nusbaum, Nicole King

**Affiliations:** 1 Department of Molecular and Cell Biology, University of California Berkeley, Berkeley, CA 94720, USA; 2 Broad Institute of MIT and Harvard, Cambridge, MA 02141, USA; 3 Department of Computational Biology, Genentech, 1 DNA Way, South San Francisco, CA 94080, USA; 4 Department of Entomology, University of Illinois at Urbana-Champaign, Urbana, IL 61801, USA; 5 Departement de Biochimie, Universite de Montreal, Montreal, Quebec, Canada

## Abstract

**Background:**

Metazoan multicellularity is rooted in mechanisms of cell adhesion, signaling, and differentiation that first evolved in the progenitors of metazoans. To reconstruct the genome composition of metazoan ancestors, we sequenced the genome and transcriptome of the choanoflagellate *Salpingoeca rosetta*, a close relative of metazoans that forms rosette-shaped colonies of cells.

**Results:**

A comparison of the 55 Mb *S. rosetta *genome with genomes from diverse opisthokonts suggests that the origin of metazoans was preceded by a period of dynamic gene gain and loss. The *S. rosetta *genome encodes homologs of cell adhesion, neuropeptide, and glycosphingolipid metabolism genes previously found only in metazoans and expands the repertoire of genes inferred to have been present in the progenitors of metazoans and choanoflagellates. Transcriptome analysis revealed that all four *S. rosetta *septins are upregulated in colonies relative to single cells, suggesting that these conserved cytokinesis proteins may regulate incomplete cytokinesis during colony development. Furthermore, genes shared exclusively by metazoans and choanoflagellates were disproportionately upregulated in colonies and the single cells from which they develop.

**Conclusions:**

The *S. rosetta *genome sequence refines the catalog of metazoan-specific genes while also extending the evolutionary history of certain gene families that are central to metazoan biology. Transcriptome data suggest that conserved cytokinesis genes, including septins, may contribute to *S. rosetta *colony formation and indicate that the initiation of colony development may preferentially draw upon genes shared with metazoans, while later stages of colony maturation are likely regulated by genes unique to *S. rosetta*.

## Background

Metazoan multicellularity and development are rooted in basic mechanisms of cell adhesion, signaling, and differentiation that were present in the unicellular and colonial progenitors of metazoans. Reconstructing the evolution of metazoans from their single celled ancestors promises to illuminate one of the major transitions in evolutionary history, while also revealing fundamental mechanisms underlying metazoan cell biology and multicellularity. Although the first metazoans evolved over 600 million years ago, insights into their biology and origin may be gained through the comparison of metazoan genomes with those of their closest living relatives, the choanoflagellates [1-3]. Indeed, the genome of the first sequenced choanoflagellate, the single-celled species *Monosiga brevicollis*, provided evidence that diverse protein domains characteristic of metazoan signaling and adhesion proteins (for example, tyrosine kinase (TK), cadherin, and Hedgehog (Hh) domains) evolved before the divergence of choanoflagellates and metazoans [2].

**Figure 1:**
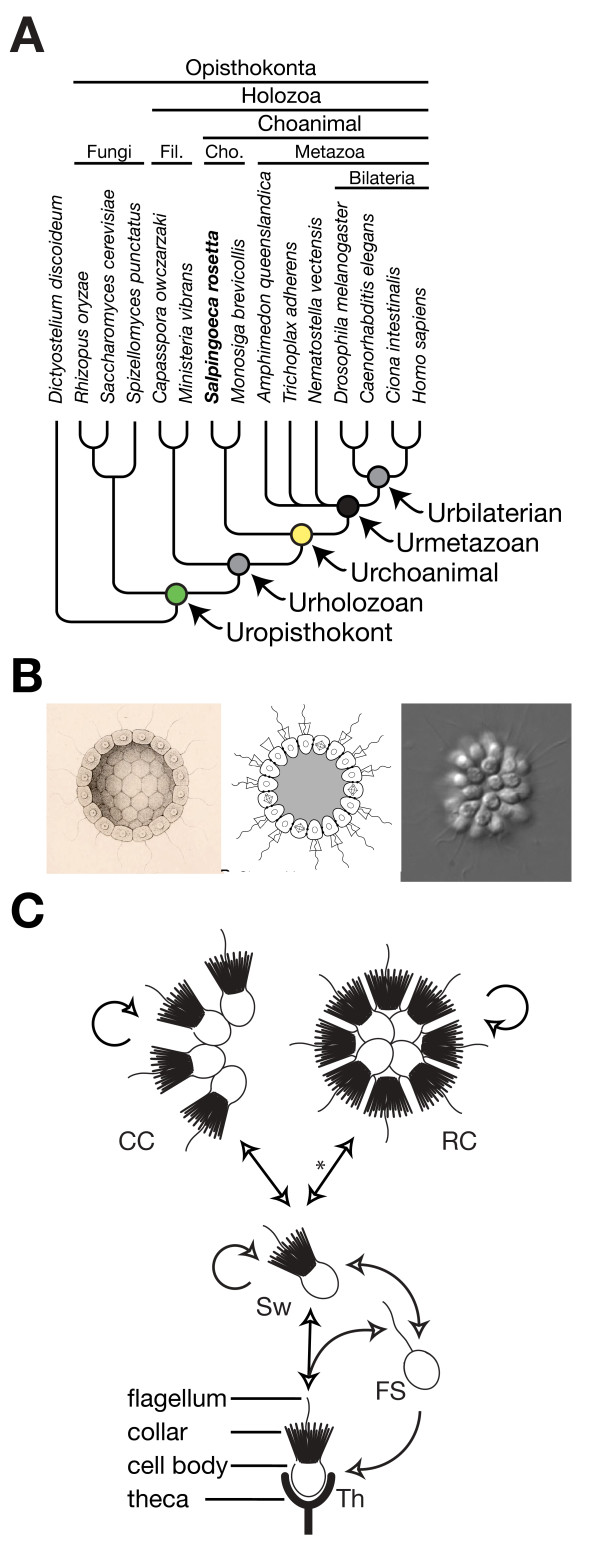
***Salpingoeca rosetta *as a model for studying the ancestry of metazoan multicellularity**. **(a) **Choanoflagellates are the closest living relatives of Metazoa [1,3,68]. Taxonomic groupings are indicated above the phylogeny and the last common ancestors of each group are indicated as colored circles at nodes. The topology of the reference phylogeny was based on a consensus of results from [68-70]. Branches were collapsed at the base of Metazoa to reflect current uncertainty about the identity and branch order of the most basal metazoan phyla [69,71-73]. The black, yellow, and green colored nodes are used in both Figures 1 and 2 to represent the Urmetazoan, Urchoanimal, and Uropisthokont, respectively. **(b) **The evolution of metazoans from their single-celled ancestors is hypothesized to have involved a transition through a simple colonial form, such as Haeckel's Blastea (left, from Figure 117 of [4]) or Nielsen's Choanoblastea (center, from [5]), that resembles the rosette colonies formed by *S. rosetta *(right). **(c) ***S. rosetta *can transition through at least five morphologically and behaviorally differentiated cell types [6]. Solitary 'thecate' cells attached to a substrate (Th) can produce solitary swimming (Sw) cells or solitary fast swimming (FS) cells, either through cell division or theca abandonment. Solitary swimming cells can divide completely to produce solitary daughter cells or remain attached after undergoing incomplete cytokinesis to produce either chain colonies (CC), or rosette colonies (RC) in the presence of the bacterium *Algoriphagus machipongonensis *(asterisk) [6,18,64]. Fil., Filasterea; Cho., Choanoflagellates.

The evolution of metazoans from their single-celled ancestors is hypothesized to have involved a transition through a colonial intermediate [4,5], the Urblastea, which may have been composed of choanoflagellate-like cells [5] (Figure 1). The rosette-shaped colonies formed by the choanoflagellate *Salpingoeca rosetta *evoke the hypothesized Urblastea (Figure 1a, b). In addition to rosette colonies, the life history of *S. rosetta *includes diverse cell types and morphologies, including linear chains of cells ('chain colonies'), slow and fast swimmer cells, and thecate cells that attach to substrates through a secreted structure called a theca (Figure 1c) [6]. The diversity of these forms is comparable to the number of cell types observed in sponges and placozoans [7]. Therefore, sequencing the *S. rosetta *genome would provide an opportunity to investigate how genome evolution and cell differentiation in the ancestors of metazoans and multicellular choanoflagellates laid the foundations for metazoan cell biology and development. Furthermore, comparisons between the genomes of *M. brevicollis *and *S. rosetta *offer the opportunity to investigate the genetic bases of multicellularity in choanoflagellates. To these ends, we have sequenced and analyzed the *S. rosetta *genome and transcriptome during multiple key phases in the *S. rosetta *life history.

## Results and discussion

The approximately 55 Mb *S. rosetta *genome was sequenced to 33× average coverage with a combination of Sanger and 454 technology and assembled into 154 scaffolds with an N50 average length of 1.52 Mb (Table S1 in Additional file 1). The genome assembly is largely complete, capturing approximately 96% of transcripts assembled *de novo *from RNA-seq data (Table S2 in Additional file 1). Predicted telomeres were found at both ends of 21 scaffolds and 24 additional scaffolds contain a single telomeric end, suggesting that *S. rosetta *has a minimum of 33 chromosomes (Table S3 in Additional file 1). A starting set of *ab initio *gene predictions generated by the Broad Institute annotation pipeline trained with ESTs (generated by Sanger chemistry) was refined using 21 Gb of transcriptome sequence (generated by Illumina chemistry) collected from diverse life history stages (Additional file 1, Figure S1). This gene catalog contains 11,629 genes, of which 98% are supported by transcriptome sequence data (Table S1 in Additional file 1). Aligning the protein sequences from this gene set to the *M. brevicollis *protein set revealed 4,994 orthologous pairs, yet the two species display relatively little gene synteny (Figure S14 in Additional file 1).

**Figure 2:**
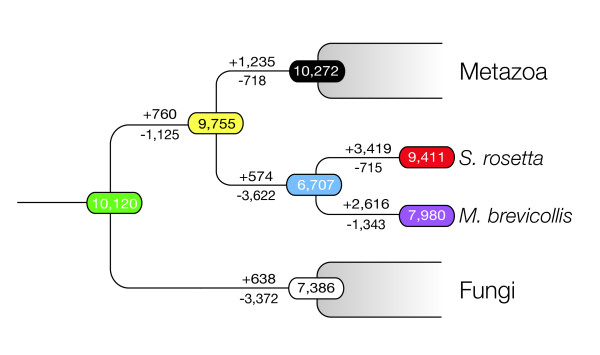
**Gene gains and losses preceding the origin of metazoans**. Reconstruction of ancestral gene content (predicted number of ortholog clusters indicated at each node) reveals that metazoans evolved as many as 1,235 ortholog clusters since their divergence from choanoflagellates and as many as 1,995 following their divergence with fungi, off-setting the loss of 20% of the Uropisthokont gene content. In contrast, current genome data suggest that the choanoflagellate and fungal lineages were dominated by gene loss. Choanoflagellates lost 47% of the Uropisthokont gene content and 37% of the gene content present in the Urchoanimal. Similarly, the fungal linage has lost 33% of the Uropisthokont gene content. Predicted ortholog cluster gain (+) and loss (-) is indicated, respectively, above and below each branch. Colored ovals represent predicted ancestral ortholog cluster content: red, *S. rosetta*; purple, *M. brevicollis*; black, Urmetazoan; white, Urfungus; blue, Urchoanoflagellate; yellow, Urchoanimal; green, Uropisthokont (Additional files 4 and 5).

To reconstruct the gene contents of the progenitors of metazoans and choanoflagellates, we compared the genomes of *S. rosetta *and *M. brevicollis *[2] with the sequenced genomes of 32 representative metazoans and metazoan outgroups (Table S4 in Additional file 1). Evolutionary relationships among genes from different genomes were predicted using OrthoMCL2 [8] to identify 'ortholog clusters' (Additional files 2 and 4). The 11,629 genes of *S. rosetta *fall into 9,411 ortholog clusters (that is, some ortholog clusters contain multiple *S. rosetta *genes). The evolutionary history of each ortholog cluster was inferred by mapping its distribution onto a reference phylogeny (Figure 1a), allowing us to gain insight into the composition of ancestral genomes and patterns of gene gain and loss in the lineages leading to metazoans, choanoflagellates, and fungi (Figure 2; Additional file 5). Gene families and protein domains of particular interest were also curated manually (see, for example, Figures S7 to S9, S13 and Tables S6 and S8 in Additional file 1).

The genomes of the ancestors ('Ur-'; Figure 1a) of metazoans and opisthokonts (metazoans + choanoflagellates + fungi) were each predicted to have contained members of about 10,000 ortholog clusters. While nearly 20% (1,843) of the ortholog clusters from the Uropisthokont were lost along the lineage leading to the Urmetazoan, this lineage also experienced an equivalent amount of gene gain (Figure 2). In contrast, fungi and choanoflagellates apparently lost representation from 33% (3,372) and 47% (4,747) of the ancestral Uropisthokont ortholog clusters, respectively, but experienced only half as much gene gain. The *S. rosetta *genome also reveals that *M. brevicollis *has lost an additional 1,343 genes. Therefore, the *S. rosetta *genome sequence substantially clarifies the gene content of the Urchoanimal. Future sequencing of additional choanoflagellate genomes will further refine inferences about the gene content of the Urchoanimal, presumably by reducing the number of genes thought to be metazoan. Nonetheless, patterns of gene gain and loss based on currently sequenced genomes speak to the richness of the gene complement in the Uropisthokont [3] and emphasize the role that gene birth may have played in the evolution of biological novelties such as metazoan multicellularity.

Therefore, we next characterized the 5,706 ortholog clusters that appear to have evolved along the stem lineage leading to metazoans (Additional file 3). These metazoan-specific ortholog clusters include homologs of genes that regulate cell adhesion, including δ-catenin and β-laminin, as well as genes involved in the transforming growth factor (TGF)-β and Wnt developmental signaling pathways. Many of the core components of the TGF-β and Wnt signaling pathways (for example, TGF-β, TGF-β receptor, Smad, Wnt, Wntless, β-catenin, and TCF) were identified in every metazoan genome included in our analysis, underscoring their early evolution and fundamental importance to metazoan biology.

Genes shared between choanoflagellates and metazoans were present in the progenitors of metazoans and may have contributed to the genomic foundations of the origin of metazoans. We find that the evolution of the monophyletic 'Choanimal' clade (which contains choanoflagellates and metazoans, and is not to be confused with the paraphyletic 'Choanozoa' (see Endnote a)), was marked by a disproportionate gain of genes with Gene Ontology terms [9] for metazoan cell adhesion and cell-junction organization (Table S5 in Additional file 1), including cadherins, PATJ (a component of adherens junctions) and KANK/vab-19 (an ankyrin repeat protein required for proper embryonic epidermal elongation and muscle attachment to the epidermis in *Caenorhabditis elegans *[10]). Ortholog clusters involved in metazoan neuropeptide signaling and glycosphingolipid metabolism also increased in abundance (Table S5 in Additional file 1). In addition, the *S. rosetta *genome, like that of *M. brevicollis*, contains a diverse and abundant repertoire of TKs (see Endnote b) [2,11,12] (Additional file 6). Ninety percent of the *S. rosetta *cytoplasmic TKs are conserved in the *M. brevicollis *genome, and *S. rosetta *has homologs of two adhesion-associated cytoplasmic TKs, FAK and Fer, that were apparently lost in *M. brevicollis *(Table S6 in Additional file 1). In contrast, only 21% of receptor TKs (RTKs) from *S. rosetta *and *M. brevicollis *form orthologous pairs. The added sequence diversity provided by the *S. rosetta *genome also revealed that choanoflagellates may have divergent homologs of metazoan Eph RTKs that were not originally detected in the *M. brevicollis *genome. Eph RTKs are key regulators of cell migration during development, regulating cellular organization through differential cell repulsion and adhesion [13]. Their discovery in *S. rosetta *lays the foundation for investigating core and ancestral functions of these important receptors.

The *S. rosetta *genome now provides a platform for investigating the regulation of cell differentiation in choanoflagellates and the potential evolutionary connections between the cell biology of choanoflagellates and metazoans. Therefore, we analyzed the transcriptional profiles of samples enriched in each of four different *S. rosetta *cell types: thecate cells, swimming cells (a mix of slow and fast swimmers), chain colonies, and rosette colonies (Figure 1c; Figure S1 in Additional file 1; Additional file 7). Using three independent analytical approaches we identified 480 *S. rosetta *genes that were consistently upregulated in colonies (chains and rosettes) compared to solitary cells (swimming and thecate) and 1,410 genes that were consistently upregulated in thecate cells relative to swimming solitary cells and colonies (Figures S2, S3 and S4 in Additional file 1; Additional files 8 to 13). For example, in colonies and thecate cells distinct subsets of TKs, cadherins (notable for their roles in metazoan cell signaling and adhesion) and Hh-domain containing proteins (see Endnote c) were significantly upregulated (Figures S5, S6, and S12 in Additional file 1), although their functions in these contexts are unknown.

**Figure 3:**
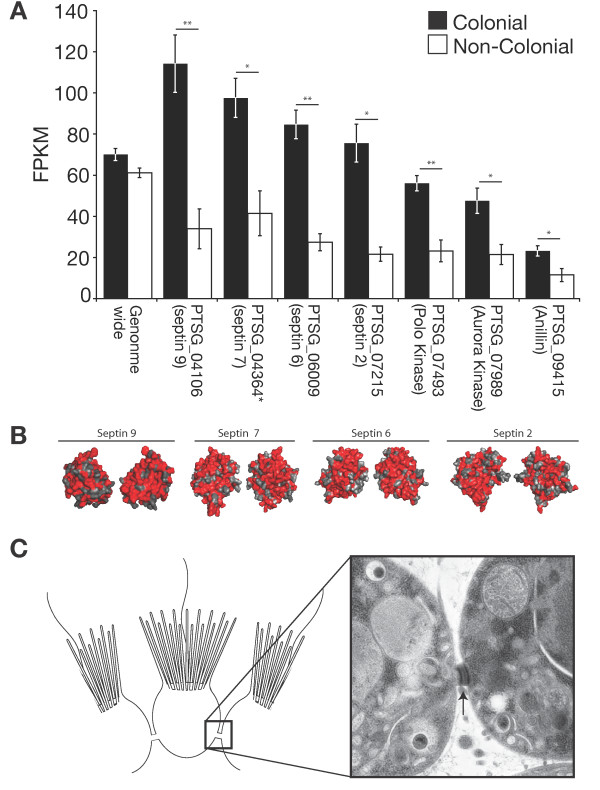
**Septins are upregulated in colonial cells**. **(a) **Unlike the average for all genes in the genome, septins, the septin-associated Polo and Aurora kinases, and Anillin are each significantly upregulated in colonial cells. FPKM, fragments per kilobase per million reads. Error bars are standard error: *P < 0.05, **P < 0.01. **(b) **Conserved and similar residues shared between *S. rosetta *septins and human septins (red) on monomer surfaces predicted to interact in human septin filaments [74] suggest that *S. rosetta *septins also form filaments. **(c) **Septins regulate cytokinesis in metazoans and fungi [14,16], providing a potential connection to the narrow intercellular bridges (arrowhead), likely formed through incomplete cytokinesis, that connect neighboring cells in *S. rosetta *colonies.

Perhaps most illuminating was the observation that all four members of the *S. rosetta *septin gene family were significantly upregulated in colonies (Figure 3a). Septins, conserved GTPases that regulate cytokinesis in fungi and metazoans, were first identified in yeast through a screen for cytokinesis defects; septin mutants frequently failed to undergo proper cytokinesis and therefore exhibited multicellular phenotypes [14]. Metazoan septins also stabilize intercellular bridges such as midbodies and ring canals [15,16]. During metazoan cytokinesis and in intercellular bridges, a set of specific septin monomers polymerize to form cytoskeletal filaments [17]. The four *S. rosetta *septins have conserved amino acid residues on predicted interacting surfaces, suggesting they may also form filaments (Figure 3b; Figures S7 and S8 in Additional file 1). Interestingly, *S. rosetta *homologs of other midbody-associated proteins and septin regulators, including Aurora kinase, the scaffolding protein Anillin, and Polo kinase, are also significantly upregulated in colonies (Figure 3a). The coordinated upregulation of septins and septin regulators is notable because colony development in *S. rosetta *occurs by incomplete cytokinesis, such that neighboring cells remain physically linked by intercellular bridges (Figure 3c) [6,18].

**Figure 4:**
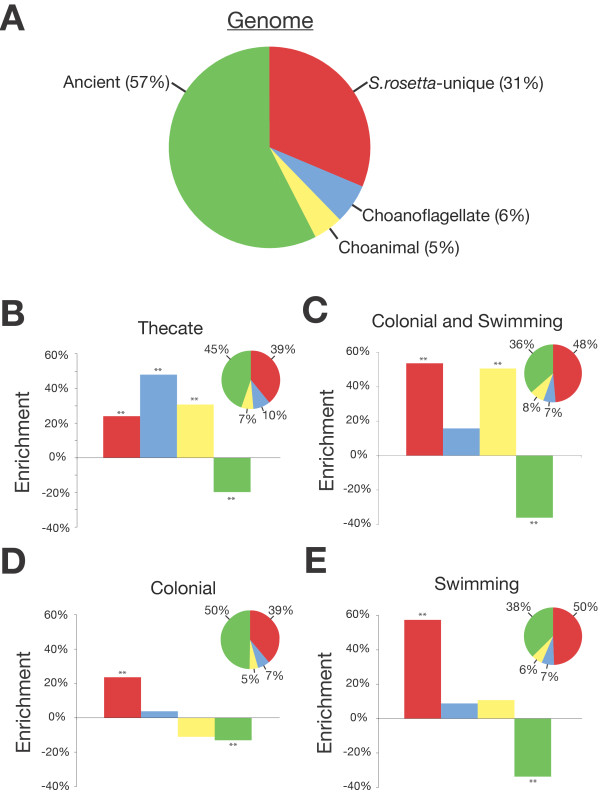
**Different *S. rosetta *cell types disproportionately upregulate genes with different evolutionary histories**. **(a) **The majority (57%) of *S. rosetta *genes are ancient and evolved prior to the divergence of choanoflagellates, metazoans and fungi. An additional 5% of *S. rosetta *genes emerged along the stem lineage leading to *S. rosetta *and metazoans and 6% evolved along the choanoflagellate stem lineage. Thirty-one percent of genes in the *S. rosetta *genome are apparently unique to *S. rosetta*. **(b-e) **The evolutionary history of *S. rosetta *genes upregulated in different cell types (pie charts) and the percent enrichment (y-axis) relative to the *S. rosetta *genome (bar graphs). Color code: red, *S. rosetta*-specific genes; blue, genes restricted to choanoflagellates; yellow, genes uniquely shared by choanoflagellates and metazoans; green, genes restricted to opisthokonts. (b) Thecate cells. (c) Colonies and swimming cells. (d) Colonies (rosettes and chains). (e) Swimming cells. **P < 0.01.

The genes that regulated cell differentiation in the progenitors of metazoans may have provided the foundations for the spatiotemporal regulation of cell differentiation that underpins metazoan development. Therefore, understanding the evolutionary history of genes differentially expressed in different *S. rosetta *cell types may suggest which cell types are most conducive to the study of metazoan origins. Of the 11,628 genes in the *S. rosetta *genome, at least 57% were present in the Uropisthokont, 5% arose on the Urchoanimal stem, 6% are choanoflagellate-specific, and 31% are apparently unique to *S. rosetta *(Figure 4a; Table S7 in Additional file 1). The evolutionary histories of genes upregulated in specific cell types deviated significantly from this distribution. For example, thecate cells disproportionately upregulated genes that evolved within choanoflagellates, after their divergence from the metazoan stem lineage (Figure 4b; Table S7 in Additional file 1). Therefore, the unusual morphology and transcriptional profile of thecate cells suggest that important aspects of their biology may be unique to choanoflagellates. Colony development, in contrast, has potential relevance for understanding the regulation of early metazoan multicellularity. Colonies develop from a subset of solitary swimming cells [6], so the most likely regulators of colony development are the 352 genes that are specifically upregulated in both solitary swimming cells and in colonies (Figure 4c; Table S7 in Additional file 1). Interestingly, this set is highly enriched in genes that are exclusively shared with metazoans and that presumably evolved along the Urchoanimal stem lineage. Genes involved in the maintenance of mature colonies, as opposed to those involved in regulating early colony development, would be expected to be specifically upregulated in colonies (Figure 4d; Table S7 in Additional file 1), but not in the single cells from which they develop (Figure 4e; Table S7 in Additional file 1). This set was enriched in genes unique to *S. rosetta*. Taken together, these data led us to hypothesize that the initiation of *S. rosetta *colony development draws upon genes shared with metazoans, while later stages of colony maturation are regulated by genes that are unique to *S. rosetta*.

## Conclusions

Although the progenitors of metazoans expired over 600 million years ago [19,20], genome comparisons between metazoans and their closest relatives, the choanoflagellates, can offer detailed insights into the evolutionary foundations of metazoan genomes and gene families [20]. The *S. rosetta *genome refines the catalog of metazoan-specific genes and highlights the potential relevance of key gene families to the evolution of defining features of metazoan biology. Genes with a variety of evolutionary histories - including highly conserved genes with functions that are integral to eukaryotic cell biology, genes that evolved before the choanimals and that were subsequently co-opted to new metazoan-specific functions, and new genes whose evolution may have served as key innovations - shaped the evolution of metazoans from their protistan ancestors [21,22]. With this more complete gene catalog, it is now possible to reconstruct the ancestry of metazoan gene families in unprecedented detail (for example, Figures S6 and S9 in Additional file 1, and [23,24]). The *M. brevicollis *genome sequence previously revealed that diverse protein domains in metazoan signaling and adhesion genes, including cadherin, Hh, and TK, evolved before the origin of metazoan multicellularity [2]. The *S. rosetta *genome now reveals that the Urchoanimal genome contained representatives of at least eight metazoan TK gene families (Table S6 in Additional file 1), including the developmentally important Eph RTKs, and raises questions about their ancestral functions in the Urchoanimal [25,26].

In addition to expanding gene family representation, the *S. rosetta *genome also sheds light on the pathways in which these genes are traditionally thought to operate. For example, while some components of the TK and Hh developmental signaling pathways are conserved in choanoflagellates (for example, Src, Eph RTK, and Patched), others are not. Therefore, the evolution of these pathways along the metazoan stem lineage likely involved an as-yet undefined combination of protein domain shuffling, gene cooption, and evolution of new protein-protein interactions [2,27] that promises to be further elucidated by the continued study of diverse early-branching metazoans, choanoflagellates and other metazoan outgroups [24,28]. In contrast, components of the Wnt pathway have not been identified in any sequenced non-metazoan genome, including those of *S. rosetta *and *M. brevicollis*, suggesting that the pathway did not evolve until after the divergence of metazoans and choanoflagellates [29-31]. A sponge classical cadherin has proven capable of binding *in vitro *[23] to its cognate β-catenin, a Wnt pathway effector, suggesting that at least a portion of the critical interactions in the Wnt pathway evolved before the Cambrian radiation. Given the ubiquity of Wnt pathway components in metazoans and their essential roles in regulating embryonic patterning in diverse animals, it is therefore possible that the evolution of the Wnt pathway was critical to the early evolution of metazoans.

Finally, the *S. rosetta *genome offers the opportunity to investigate whether choanoflagellate colony formation and metazoan development are regulated by conserved mechanisms. The upregulation of conserved genes and gene families in colonies, such as septins, cadherins, and Hh-related proteins, is intriguing and warrants further investigation to fully understand their current and ancestral functions. Taken together, the *S. rosetta *genome and transcriptome suggest that the genome of the last common ancestor of choanoflagellates and metazoans contained genes and domains that orchestrate development in modern animals but underwent important changes in gene content and regulation *en route *to the evolution of the first metazoan. Further refinement of ancestral genomes through comparative genomics with additional choanoflagellate genomes and functional efforts in choanoflagellates and sponges promises to reveal the minimal set of genes required for metazoan development and multicellularity.

## Materials and methods

### *Salpingoeca rosetta *culture conditions

*S. rosetta*, a colonial choanoflagellate originally isolated from Hog Island, Virginia, was cultured with co-isolated bacteria at 25°C in natural seawater infused with cereal grass media [32]. The strain sequenced in this study is deposited at the ATCC under strain number ATCC PRA-366.

### Isolation of *S. rosetta *genomic DNA

Genomic DNA was harvested from a monoxenic culture of *S. rosetta *in which the sole source of bacteria was *Algoriphagus machipongonensis *[6]. *S. rosetta *DNA was separated from the *A. machipongonensis *DNA on a CsCl gradient as described for the genome sequencing of *M. brevicollis *[2].

### Genome sequencing

Purified *S. rosetta *genomic DNA was sequenced with 454 and Sanger Whole Genome Shotgun methodology as described below.

#### 454 sequencing

454 fragment and approximately 3 kb jumping libraries were generated as previously described [33]. In short, *S. rosetta *genomic DNA was sheared into small fragments, approximately 600 bp for fragment and approximately 3 kb for jumping libraries. For fragment library construction DNA was ligated on both ends with 454 sequencing adapters. For 3 kb jumping library construction, DNA was ligated with biotinylated adapters on both ends to facilitate circularization. Adapted DNA was circularized, sheared and resulting fragments were ligated on both ends with 454 sequencing adapters. Library fragments containing biotin were retrieved using streptavidin beads. Both library types were subjected to emulsion PCR and sequenced with approximately 400 base titanium chemistry reads using a 454 GS FLX instrument following the manufacturer's recommendations (454 Life Sciences/Roche, Branford, Connecticut, USA).

#### Sanger sequencing

Genomic DNA was sheared and cloned into plasmid (4 kb and 10 kb insert) and Fosmid (40 kb) vectors using standard methods. Resulting whole genome shotgun libraries were paired-end Sanger sequenced using standard methods.

### Genome assembly

454 data were first assembled using 454's Newbler assembler [34]. 454 assembly was then combined with Sanger data using the HybridAssemble [35] module of the ARACHNE assembler [36]. The assembly was then manually modified to close additional gaps and break misassembled joins using ARACHNE tools.

### Telomere identification

Six supercontigs containing telomeric ends (Table S3 in Additional file 1, fourth column) were identified by searching the genome assembly for TTAGGG repeats. Examination of the subtelomeric regions of these six supercontigs did not reveal genes that are shared at the other telomeres below, so they appear to be aberrant or newly formed telomeres without the subtelomeric repeated regions of most telomeres.

Additional telomeric supercontigs were identified by searching the raw reads from the approximately 40 kb insert fosmids with 1,000 bases of TTAGGG repeats. The mate pairs of the first 250 such hits, all of which were in plus/minus arrangement, indicating that they were from telomeres, were then searched against the supercontigs to identify telomeric supercontigs. This search revealed 41 such supercontigs (fifth column of Table S3 in Additional file 1), including four of the six with assembled TTAGGG repeats. Clearly this is an underestimate of the number of telomeres, both because only four of the six assembled ones were identified, and because this search yields a Poisson distribution of such hits (fifth column of Table S3 in Additional file 1), ten of which were only hit once. From the average positions of the mate-pair hits within each supercontig it was possible to estimate the length of DNA missing between the assembly and the TTAGGG repeats of the telomere, and this is shown in the sixth column of Table S3 in Additional file 1.

Examination of these 37 telomeric supercontigs without assembled TTAGGG repeats revealed that all but a few of them have regions repeated on most of the others (seventh column in Table S3 in Additional file 1). The few exceptions are instances where the gap between the end of the supercontig and the TTAGGG repeats is near the 40 kb insert size of the fosmids, so presumably the shared subtelomeric regions are within this missing part. This approach allowed us to discover 22 additional telomeric supercontigs.

### Genome annotation

Protein-coding genes were initially annotated using a combination of *ab initio *predictions (GeneMark.hmm-ES, AUGUSTUS, GlimmerHMM), protein sequence homology-based evidence (blast, GeneWise), and transcript structures built from ESTs using the PASA package [37]. The package EVM (EVidenceModeler) [38] was used to build gene models from all available input evidence. The obtained gene models were further improved by incorporating RNAseq data from eight different conditions using PASA and inchworm pipelines to get a final gene set [39,40]. Gene models were also annotated with gene ontology terms using Blast2Go (Additional file 14) and interPro2GO (Additional file 15), and gene ontology enrichment was measured with Ontologizer 2.0 using default settings correcting for multiple testing.

### Synteny analysis

Protein sequences from *S. rosetta *and *M. brevicollis *were aligned using BLAST [41].

Best reciprocal BLAST pairs with a score cutoff of 75 were considered orthologs.

Predicted protein orthologs were mapped back to their genomic loci using BLAT [42] and plotted against the scaffolds with R to investigate synteny between the *S. rosetta *and *M. brevicollis *genomes.

### Tyrosine kinase annotation

Manual annotations for the *S. rosetta *kinases were made through BLAST [41], multiple sequence alignments, hidden Markov models, presence or absence of accessory domains and phylogenetic trees. *S. rosetta *kinases were compared to nine previously annotated kinomes: *Homo sapiens *[43], *Mus musculus *[44], *Strongylocentrotus purpuratus *[45], *Drosophila melanogaster *[46], *C. elegans *[47], *Amphimedon queenslandica *[30], *Monosiga brevicollis *[48], *Saccharomyces cerevisiae *[49], and *Selaginella moellendorffii *[50].

### Septin characterization

The final gene predictions for the *S. rosetta *genome included five septin domain encoding genes (PTSG_04106, PTSG_06009, PTSG_07215, PTSG_04363 and PTSG_04364) as predicted by Pfam [51]. A gap in the assembly suggested that PTSG_04363 and PTSG_04364 might be one gene. PCR amplification from a *S. rosetta *cDNA library using specifically designed primers (5'TCAACGAAACGATTTCAAGC and 5'GTGGTCCGAGTTGTCGACTT) confirmed this and the two gene models were merged into a new gene model (PTSG_ 04364*) (Figure S7 in Additional file 1). Conserved septin-specific residues, including the amino-terminal polybasic region, were identified manually while coiled-coil domains were predicted using the COILS program using the default settings [52]. Sequences with average probabilities below 0.8 were not considered to have coiled-coil domains (Figure S8 in Additional file 1).

### Septin structure prediction

The structure of each *S. rosetta *septin was predicted using LOOPP (version 4.0) available through the University of Texas [53,54]. Individual *S. rosetta *septin structures were loaded into MacPymol [55] and similar residues determined using NCBI BLAST [41] alignment and colored red. Each structure was then aligned to the crystal structure of the human septin filament (accession 2QAG in the Protein Data Bank).

### Phylogenetic analyses

The four *S. rosetta *septin sequences were added to a septin alignment from Momany *et al. *[56] in order to establish putative gene homology assignments (Figure S8 in Additional file 1). The sequences were aligned using the Clustal Omega multiple sequence alignment program [57] and variable sequence regions were systematically removed using Gblocks [58] with the most lenient parameters: Minimum Number Of Sequences For A Conserved Position, 81 (b1 = 81); Minimum Number Of Sequences For A Flanking Position, 81 (b2 = 81); Maximum Number Of Contiguous Nonconserved Positions, 8 (b3 = 8); Minimum Length Of A Block, 5 (b4 = 5); Allowed Gap Positions, With Half (b5 = h); Use Similarity Matrices, Yes (b6 = y); New number of positions, 210 (15% of the original 1,360 positions). A maximum likelihood analysis was performed on the resulting alignment of 183 amino acid characters using PHYML v.3.0 [59]. The WAG substitution model [60] was implemented with a mixed model of rate heterogeneity and four rate categories where the fraction of invariable sites and the gamma distribution parameter alpha were estimated from the data set. Bootstrap support (100 replicates) was estimated for the single resulting tree topology (Figure S9 in Additional file 1).

### Reconstructing gene gain and loss in opisthokonts

To characterize how gene content changed during the evolution of the opisthokonts, ortholog clusters were mapped to a reference phylogeny [61,62] using a Dollo parsimony model of evolution [63] and the minimal gene content at each node and the change along the subsequently diverging lineages was estimated.

### Cell type enrichment

Solitary swimming (Sw) cells were isolated from the supernatant fraction of cultures grown in the presence of mixed bacteria, but not *A. machipongonensis *[18].

Thecate (Th) cells were collected from cultures by removing the supernatant, washing three times with 10 ml of culture media and removing the attached cells from the plate surface with a plastic cell lifter.

Cultures consisting primarily of chain colonies (CC) were generated by diluting 2 ml of cells from the supernatant of solitary swimming (Sw) cells into 15 ml fresh medium every day for 1 to 2 weeks.

Cultures consisting primarily of rosette colonies (RC) were produced using two different strategies. In the first approach, a culture of solitary swimming (Sw) cells was inoculated with live *A. machipongonensis *bacteria [18], which induces the development of rosette colonies (RC) that became the dominant form in the culture within 2 days [6,18,64]. Rosette colonies (RC) were also isolated from cultures grown exclusively with live *A. machipongonensis *[6].

### RNAseq

Total RNA was isolated from *S. rosetta *cultures using the RNAeasy (Qiagen, Venlo, The Netherlands) kit and four consecutive rounds of oligo-dT hybridization, washing, and elution with Oligotex kit (Qiagen) were used to purify mRNA. Purified mRNA was treated with Ambion Turbo DNA-free (Life Technologies, Carlsbad, California, USA) per the manufacturer's recommendation. The integrity of the mRNA was assessed using an Agilent 2100 Bioanalyzer (Agilent Technologies, Santa Clara, California, USA) and quantified using RNA Quant-it assay for the Invitrogen Qubit Fluorometer (Life Technologies, Carlsbad, California, USA).

Strand specific dUTP Illumina RNA-seq libraries were generated from 200 ng mRNA as previously described [65] with the following modifications. mRNA was fragmented in 1× fragmentation buffer (Affymetrix, Santa Clara, California, USA) at 80°C for 4 minutes, purified and concentrated to 6 μl following ethanol precipitation. Illumina sequencing adapters containing 8-base barcodes were ligated to each sample, enabling pooling of libraries. Adaptor ligation was done with 1.2 μl of barcoded Illumina adaptor mix and 4,000 cohesive end units of T4 DNA Ligase (New England Biolabs, Ipswich, Massachusetts, USA) overnight at 16°C in a final volume of 20 μl. Final library insert size ranged from 225 to 425 bp. Libraries were sequenced with 68 base paired-end reads on an Illumina GAII instrument (Illumina, San Diego, California, USA) following the manufacturer's recommendations.

### Identification of differentially expressed genes

#### Pairwise comparison

To identify genes differentially expressed in a particular cell type and control for environmental variation, we compared gene expression in different fractions of the same culture. All genes identified by this method have a statistically significant difference in at least 30% of the comparisons, with the remaining comparisons showing the same trend.

##### Colonial versus thecate

Read count was compared between samples (RCA1 versus ThA2, RCA2 versus ThA2, RCAM versus ThAM, RCAM versus ThM, CCM versus ThA2, CCM versus ThAM, CCM versus ThM) using edgeR installed under Bioconductor v2.8 and a gene was considered differentially expressed between colonial cells and attached cells if it was significantly differentially expressed (*P*-value < 0.05) in at least three comparisons and had a fold change greater than 1.5 in the remaining comparisons.

##### Colonial versus swimming

Read count was compared between samples (RCA1 versus SwM, RCA2 versus SwM, RCAM versus SwM, CCM versus SwM) using edgeR installed under R Bioconductor v2.8 [66] and a gene was considered differentially expressed between colonial cells and swimming cells if it was significantly differentially expressed (*P*-value < 0.05) in at least two comparisons and had a fold change greater than 1.5 in the remaining comparisons.

##### Attached versus swimming

Read count was compared between samples (ThA2 versus SwM, ThAM versus SwM, ThM versus SwM) using edgeR installed under R Bioconductor v2.8 [66] and a gene was considered differentially expressed between attached cells and swimming cells if it was significantly differentially expressed (*P*-value < 0.05) in at least one comparison and had a fold change greater than 1.5 in the remaining comparisons.

#### Group comparison

RNAseq read count was compared between groups of samples using edgeR installed under R Bioconductor V2.8 [66] and genes are considered differentially expressed with *P*-value < 0.05. The comparisons include: Colony versus thecate (RCA1, RCA2, RCAM, CCM versus ThA2, ThAM, ThM); Colony VS Swim (RCA1, RCA2, RCAM, CCM versus SwM); Thecate versus Swim (ThA2, ThM, ThAM versus SwM).

#### Hierarchical clustering

FPKM values (fragments per kilobase per million reads) for each gene were log2 transformed, quantile normalized, and filtered requiring Max(log2(FPKM)) - Min(log2(FPKM)) > 2. The filtered gene set was clustered hierarchically using the gplots package installed under R Bioconductor V2.8 [66], and 22 initial clusters were manually identified. Genes from these clusters were scored as colony, swimming, thecate, colony and swimming, thecate and swimming, and colony and thecate based on their expression patterns (Figure S2 in Additional file 1).

### OrthoMCL

Predicted protein sets for 34 genomes (Table S4 in Additional file 1) were generated from the longest protein greater than 30 amino acids for each gene. Then all-vs-all blastp [41] (E-value < 1E-5) was run on the filtered proteins and the OrthoMCL2 [61] pipeline was used to build ortholog clusters with default parameters.

### Reconstructing gene gain and loss in opisthokonts

To characterize how gene content changed during the evolution of the opisthokonts, ortholog clusters from OrthoMCL2, including single gene clusters, were mapped to a reference phylogeny [61,62] using a Dollo parsimony model [63]. The minimal gene content at each node and the change along the subsequently diverging lineages were then catalogued. The presence or absence of gene families and protein domains mentioned in the text were manually verified using homologs from NCBI homologene and BLASTP and tBLASTn (cutoff e10^-3^) [41].

### Ortholog cluster origin enrichment analysis

Ortholog clusters were annotated as ancient, choanimal, choanoflagellate or *S. rosetta*-unique based on the cluster member most distantly related to *S. rosetta*. The relative frequencies of phylogenic annotations were calculated for the entire *S. rosetta *genome (Figure 4a). Expression clusters were tested for phylogenic enrichment by comparing their annotation counts to frequencies for the entire genome. Annotation counts were assumed to follow a multinomial distribution, which was validated through a Monte Carlo simulation (data not shown).

A jackknifing analysis was run to test the sensitivity of phylogenic enrichment to the species included (Figure S10 in Additional file 1); 10,000 trials were run, each with a random set of species. *S. rosetta *and *M. brevicollis *were included in all trials. Each of the 32 remaining species had an 80% probability of being included in any given trial. The OrthoMCL2 algorithm was rerun for each species set to generate new clusters. Annotation frequencies were re-calculated for the entire genome and the expression clusters were tested for phylogenic enrichment.

The MCL algorithm was run an additional 19 times to test the sensitivity of the results to the inflation parameter of the MCL algorithm (Figure S11 in Additional file 1). Values for inflation ranged from 1.1 to 3. All 34 analyzed species were included.

### Data availability

Raw 454 genome sequence data have been submitted to NCBI's Short Read Archive and can be retrieved using the following accession numbers: fragment reads (SRX015529, SRX015528, SRX015527, SRX015526, SRX015525, SRX015524, SRX015523, SRX015522, SRX015521, SRX015515, SRX015514, SRX015512, SRX015511, SRX015503, SRX015502, SRX015499, SRX015498, SRX015486, SRX015485, SRX015484, SRX015483, SRX015482, SRX015457, SRX015456); and 2 to 3 kb jumping reads (SRX015508, SRX015505, SRX015501, SRX015464, SRX015463, SRX015458). Raw Sanger sequence data have been submitted to NCBI's Trace Archive and can be retrieved using the following search parameters: CENTER_NAME = "BI" and CENTER_PROJECT = "G1237". The genome assembly was submitted to NCBI with accession number ACSY00000000. Genome sequence and transcriptome sequence have been deposited in GenBank under project codes PRJNA37927 and SRP005692, respectively. A genome browser is available at the Broad Institute website [67].

Raw sequence data from Illumina sequencing of cell-type enriched transcriptomes has been submitted to NCBI's Short Read Archive using the following accession numbers: RCAM, SRX042054 (NK96-sup - culture enriched for colonial cells grown in the presence of mixed bacterial prey and *A. machipongonensis*); SwM, SRX042053 (Col-sup - solitary swimming cells grown in the presence of mixed bacterial prey); ThA2, SRX042052 (Pxl-att - culture enriched for solitary attached cells grown only in the presence of *A. machipongonensis*); ThAM, SRX042051 (NK96-att - culture enriched for solitary attached cells grown in the presence of mixed bacterial prey and *A. machipongonensis*); ThM, SRX042050 (Col-att - culture enriched for solitary attached cells grown in the presence of mixed bacterial prey); RCA1, SRX042049 (colonies - culture enriched for colonial cells grown only in the presence of *A. machipongonensis*); CCM, SRX042047 (Chains - culture enriched for chain cells grown with mixed bacterial prey); RCA2, SRX042046 (Pxl-sup - culture enriched for colonial cells grown only in the presence of *A. machipongonensis*).

## Supplementary Material

Additional file 1 **Figures S1 to S14 and Tables S1 to S8**. Figure S1: transcriptional profiling experimental design. Figure S2: differentially expressed genes identified by hierarchical clustering. Figure S3: identification of upregulated genes. Figure S4: gene expression correlates with cell type. Figure S5: *S. rosetta *cadherin expression. Figure S6: Hedgehog signal domain-encoding genes are upregulated in thecate and colonial cell types. Figure S7: protein domain architecture of *S. rosetta *septins. Figure S8: septin sequence conservation. Figure S9: septin gene family phylogeny. Figure S10: ortholog cluster origin enrichment is robust to species composition. Figure S11: ortholog cluster origin enrichment is robust to changes in MCL (Markov Cluster algorithm) species inflation value. Figure S12: expression levels of receptor tyrosine kinase families. Figure S13: the phylogenetic distribution of important metazoan development genes or domains. Figure S14: synteny between the *S. rosetta *and *M. brevicollis *genomes. Table S1: *S. rosetta *and *M. brevicollis *genome statistics. Table S2: mapping of *de novo *transcript assembly. Table S3: telomeres predicted in the *S. rosetta *genome. Table S4: genomes used for comparative genomics. Table S5: Gene Ontology enrichment of novel genes. Table S6: *S. rosetta *tyrosine kinases. Table S7: phylogenetic distribution of genes upregulated in different cell types. Table S8: genes missing from choanoflagellate. Click here for file

Additional file 2 **Number of ortholog pairs shared between *S. rosetta *and *M. brevicollis *scaffolds**. Click here for file

Additional file 3 **The number of genes present in each of the OrthoMCL ortholog clusters**. Click here for file

Additional file 4 **The genes present in each of the OrthoMCL ortholog clusters**. Click here for file

Additional file 5 **OrthoMCL ortholog clusters predicted to be present in the ancestors reconstructed in Figure **2. Click here for file

Additional file 6 **Kinases identified in the *S. rosetta *genome**. Click here for file

Additional file 7 **Read counts and FPKM values (fragments per kilobase per million reads) for genes encoded by the *S. rosetta *genome**. Click here for file

Additional file 8 **Differentially expressed genes identified by hierarchical clustering**. Click here for file

Additional file 9 **Differentially expressed genes identified by pairwise comparison**. Click here for file

Additional file 10 **Differentially expressed genes identified by group comparison**. Click here for file

Additional file 11 **Genes identified as upregulated in colonial cells**. Click here for file

Additional file 12 **Genes identified as upregulated in thecate cells**. Click here for file

Additional file 13 **Genes identified as upregulated in swimming cells**. Click here for file

Additional file 14 **Blast2GO annotation of *S. rosetta *genome**. Click here for file

Additional file 15 **Interpro annotation of *S. rosetta *genome**. Click here for file

## Abbreviations

bp: base pair; Hh: Hedgehog; RTK: receptor tyrosine kinase; TGF: transforming growth factor; TK: tyrosine kinase.

## Endnotes

^a^The term 'Choanozoa,' first coined by Shalchian-Tabrizi *et al. *[75], refers to the paraphyletic group that contains *Capsaspora owczarzaki *and choanoflagellates, but excludes metazoans.

^b^***S. rosetta *tyrosine kinases**

The *S. rosetta *genome encodes 469 protein kinases. The 376 serine/threonine kinases are generally well conserved between *M. brevicollis *and *S. rosetta *(Table S6 in Additional file 1). In contrast, the TKs seem to be more rapidly evolving and show divergent sequences and large numbers of gains and losses (Table S6 in Additional file 1). Comparison of the 93 *S. rosetta *and 135 *M. brevicollis *TKs suggests a core choanoflagellate tyrosine kinome of approximately 51 kinases, with extensive gains and some losses in the two species. The Fer and FAK kinases appear to have been lost in *M. brevicollis*, as they are found in *S. rosetta*, metazoans, and *Capsaspora owczarzaki*. Fer and FAK both regulate cell adhesion in metazoans, suggesting that *M. brevicollis *has lost some ancestral cell-adhesion functions.

The *S. rosetta *sequences allow an improved classification of both choanoflagellate tyrosine kinomes, resulting in six new families of RTKs (RTKN-T) and the creation of other families (UTKA-H) from many of the previously unique TKs. The additional sequences also indicate orthology between some choanoflagellate and metazoan RTKs: Eph RTKs are found in both species, and several other RTKs are weakly Eph-like. More tentatively, the new RTKS family may be orthologous to the insulin/IGF1R family, although it has only partial similarity to the extracellular regions of metazoan insulin receptors.

The cytoplasmic (non-receptor) TKs are evolutionarily stable between *M. brevicollis *and *S. rosetta*, with 90% of kinases in common, the only differences being one extra CTKA and FYTK in *M. brevicollis*, and the loss of FAK and Fer from *M. brevicollis*. The large family of 15 HMTKs is completely conserved between the two sequenced choanoflagellate species.

The receptor TKs are more evolutionarily dynamic, with eight families specific to *M. brevicollis*, three families specific to *S. rosetta*, and only 21% of all RTKs orthologous between the two sequenced choanoflagellates. Another eight TK families (UTKA-H) could not be classified as cytoplasmic or receptor, and the 'TK-Unique' kinases have no clear homologs between the two species.

As with other genes, we see that *S. rosetta *TKs that are conserved with *M. brevicollis *tend to be more highly expressed in attached cells, while those unique to *S. rosetta *are more highly expressed in rosette colonies (Figure S12 in Additional file 1).

^c^***S. rosetta *cadherin and hedgling protein diversity**

The *S. rosetta *genome is predicted to encode 29 proteins containing cadherin domains [23], a number that is comparable to the complement of cadherins found in the genomes of *M. brevicollis *and many animals (including 17 in *D. melanogaster *and 32 in *C. intestinalis*) [76]. While cadherins are well known for their roles in animal cell adhesion and intercellular signaling [77], their functions in choanoflagellates are unknown. Two *S. rosetta *cadherins, PTSG_06458 and PTSG_06068, are upregulated in colonies relative to single cells and an additional six are upregulated in thecate cells relative to colonies (Figure S5 in Additional file 1).

Two of the six cadherins that are upregulated in thecate cells are homologs of Hedgling. Hedgling proteins are conserved in choanoflagellates, sponges, and cnidaria, and proposed to represent evolutionary antecedents of the metazoan developmental signaling protein Hh [2,27]. Unlike canonical metazoan Hh proteins (which contain an amino-terminal Hh signal domain and a carboxy-terminal autocatalytic HINT domain), Hedgling proteins are large transmembrane proteins that contain an amino-terminal Hh domain, an adjacent VWA domain, and multiple cadherin repeats; some also contain tumor necrosis factor receptor, Furin, and epidermal growth factor domains, although these are not universally conserved [2,78]. Prior to the sequencing of the *S. rosetta *genome, the choanoflagellate Hedglings were the only known non-metazoan Hh-domain containing proteins. With the sequencing of the *S. rosetta *genome, we have discovered five additional non-Hedgling proteins that contain Hh domains. A unifying characteristic of these proteins and all Hedglings, including those from the sponge *Amphimedon queenslandica *and the cnidarian *Nematostella vectensis*, is the positioning of the Hh domain immediately adjacent to a VWA domain; this pairing may represent an ancient cassette that was also encoded by the genes from which metazoan Hh evolved. Four of the Hh signaling domain-containing proteins also have a predicted transmembrane domain and these proteins are all upregulated in thecate cells (Figure S6 in Additional file 1). The remaining three Hh domain-containing proteins are relatively small, contain an amino-terminal signal sequence, and lack a transmembrane domain; these proteins are consistently upregulated in colonial cells where they may act as secreted ligands. Like *M. brevicollis*, the *S. rosetta *genome also encodes homologs of Patched, the Hh receptor in metazoans, allowing the possibility that the Hh-Patched interaction preceded the origin of metazoans.
