# Supplementary material for: Premetazoan genome evolution and the regulation of cell differentiation in the choanoflagellate Salpingoeca rosetta
Source: Genome Biol. 2013 Feb 18;14(2):R15. doi: 10.1186/gb-2013-14-2-r15 (PMC4054682; doi:10.1186/gb-2013-14-2-r15)
Supplement: Additional file 1 — Figures S1 to S14 and Tables S1 to S8. Figure S1: transcriptional profiling experimental design. Figure S2: differentially expressed genes identified by hierarchical clustering. Figure S3: identification of upregulated genes. Figure S4: gene expression correlates with cell type. Figure S5: S. rosetta cadherin expression. Figure S6: Hedgehog signal domain-encoding genes are upregulated in thecate and colonial cell types. Figure S7: protein domain architecture of S. rosetta septins. Figure S8: septin sequence conservation. Figure S9: septin gene family phylogeny. Figure S10: ortholog cluster origin enrichment is robust to species composition. Figure S11: ortholog cluster origin enrichment is robust to changes in MCL (Markov Cluster algorithm) species inflation value. Figure S12: expression levels of receptor tyrosine kinase families. Figure S13: the phylogenetic distribution of important metazoan development genes or domains. Figure S14: synteny between the S. rosetta and M. brevicollis genomes. Table S1: S. rosetta and M. brevicollis genome statistics. Table S2: mapping of de novo transcript assembly. Table S3: telomeres predicted in the S. rosetta genome. Table S4: genomes used for comparative genomics. Table S5: Gene Ontology enrichment of novel genes. Table S6: S. rosetta tyrosine kinases. Table S7: phylogenetic distribution of genes upregulated in different cell types. Table S8: genes missing from choanoflagellate. [file gb-2013-14-2-r15-S1.DOC]

Additional file 1

**Premetazoan genome evolution and the regulation of multicellular development in the choanoflagellate *Salpingoeca rosetta***

**Authors:**

Stephen R Fairclough^1^*, Zehua Chen^2^*, Eric Kramer^3^, Qiandong Zeng^2^, Sarah Young^2^, Hugh M Robertson^4^, Emina Begovic^1^, Daniel J Richter^1^, Carsten Russ^2^, M Jody Westbrook^1^, Gerard Manning^3^, B Franz Lang^5^, Brian Haas^2^, Chad Nusbaum^2^†, Nicole King^1^†

correspondence to: [nking@berkeley.edu](mailto:nking@berkeley.edu) (N.K.), chad@broadinstitute.org (C.N.)

**This PDF file includes:**

Figures S1 to S14

Tables S1 to S8

**Table of contents:**

Figure S1. Transcriptional profiling experimental design.

Figure S2. Differentially expressed genes identified by hierarchical clustering.

Figure S3. Identification of upregulated genes.

Figure S4. Gene expression correlates with cell type.

Figure S5. *S. rosetta* cadherin expression.

Figure S6. Hedgehog signal domain-encoding genes are upregulated in thecate and colonial cell types.

Figure S7. Protein domain architecture of *S. rosetta* septins.

Figure S8. Septin sequence conservation.

Figure S9. Septin gene family phylogeny.

Figure S10. Ortholog cluster origin enrichment is robust to species composition.

Figure S11. Ortholog cluster origin enrichment is robust to changes in MCL (Markov Cluster algorithm) species inflation value.

Figure S12. Expression levels of receptor tyrosine kinase families.

Figure S13. Metazoan developmental protein domains in choanoflagellates.

Figure S14. Synteny between the *S. rosetta* and *M. brevicollis* genomes.

Table S1. *S. rosetta* and *M. brevicollis* genome statistics.

Table S2. Mapping of *de novo* transcript assembly.

Table S3. Telomeres predicted in the *S. rosetta* genome.

Table S4. Genomes used for comparative genomics.

Table S5. Gene Ontology enrichment of novel genes.

Table S6. *S. rosetta* tyrosine kinases.

Table S7. Phylogenetic distribution of genes upregulated in different cell types.

Table S8. Manual curation of gene families predicted to be absent from choanoflagellates by OrthoMCL.

**Figure S1.**

**Transcriptional profiling experimental design.** Each cell type sampled from different stages in the *S. rosetta* life history (left) was grown with either the colony-inducing bacterium *A. machipongonensis*, mixed environmental bacteria, or mixed environmental bacteria supplemented with *A. machipongonensis* (right). Experimental samples are labeled (middle) with abbreviations that denote the cell type and bacterial prey. RCA1 refers to rosette colonies fed with only *A. machipongonensis*. RCA2 and ThA2 refer to rosette colonies and thecate cells, respectively, from the same culture fed with *A. machipongonensis*. RCAM and ThAM refer to rosette colonies and thecate cells, respectively, from the same culture fed with mixed bacteria and *A. machipongonensis*. CCM refers to chain colonies fed with mixed bacteria. SwM and ThM refer to solitary swimming cells and thecate cells, respectively, from the same culture fed mixed bacteria.

**
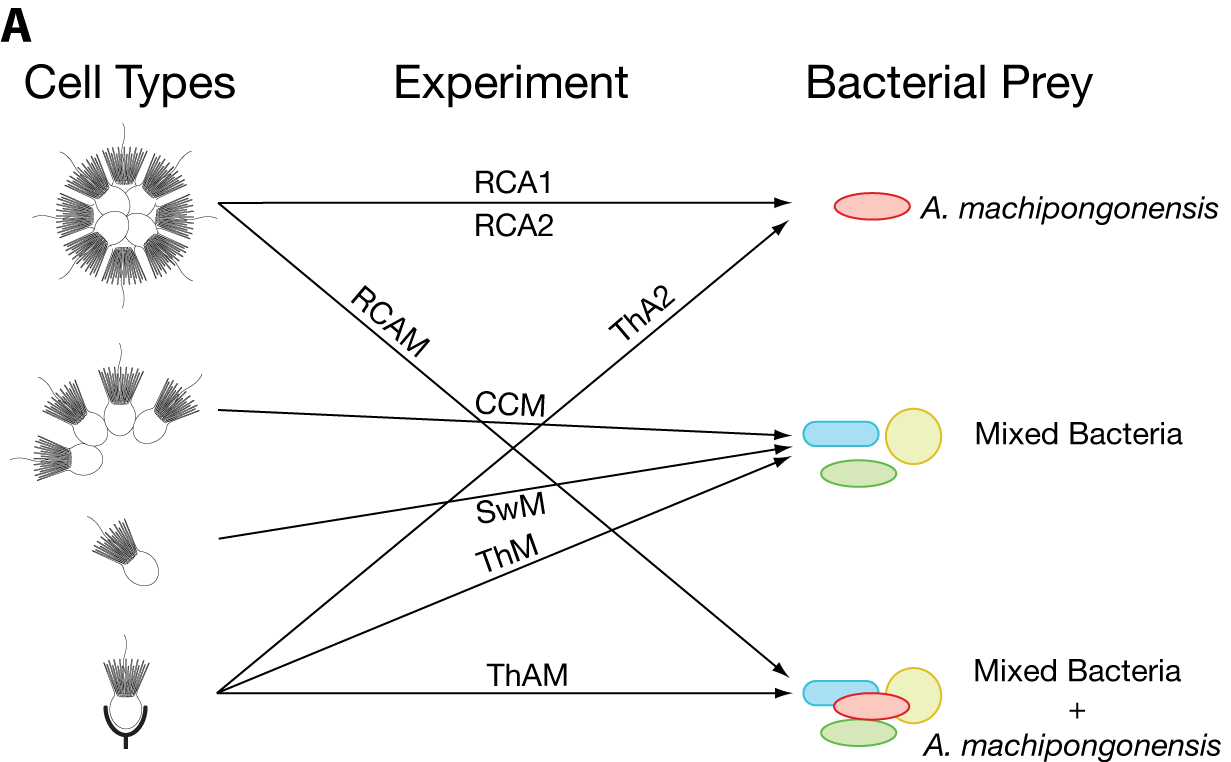
**

**Figure S2.**

**Differentially expressed genes identified by hierarchical clustering.** **A.** RNA fragment abundance (FPKM) was log2 transformed, quantile normalized, and filtered requiring Max(log2(FPKM)) - Min(log2(FPKM))>2. The filtered genes were clustered hierarchically (y-axis) as were the samples (x-axis, see Fig. S1 legend for explanation of samples) and 22 initial clusters were manually selected (numbered red boxes). Z-score reflects the number of standard deviations away from the mean. **B.** Genes from these clusters were scored as colony, swimming, thecate, colony and swimming, thecate and swimming, and colony and thecate based on their expression and placed in a final cluster (f#) (Additional file 8). Upregulated genes are displayed in yellow; downregulated genes are in blue.


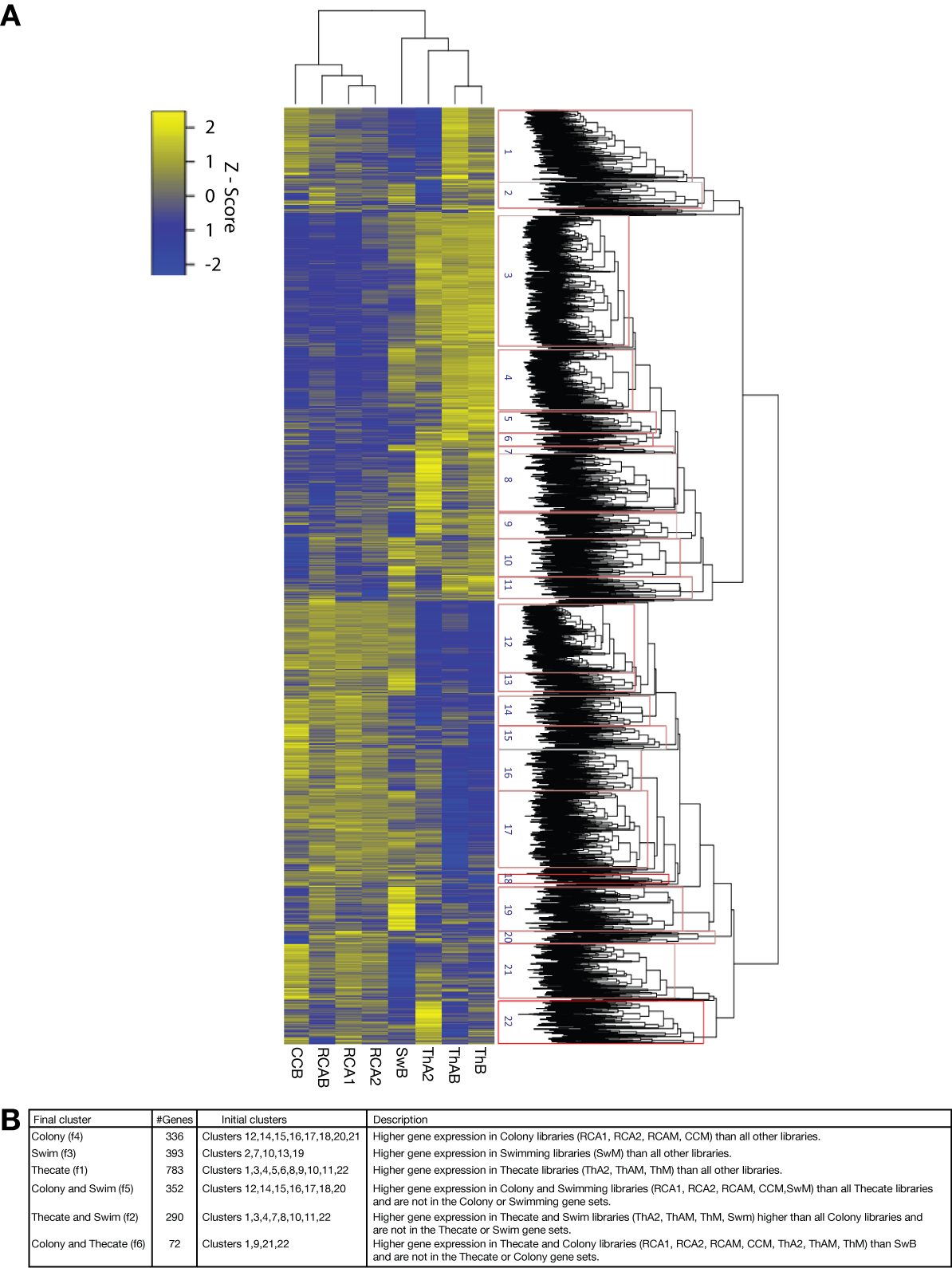


**Figure S3.**

**Identification of upregulated genes.** We identified sets of upregulated genes from cell types as follows: samples grown in the same conditions (pairwise), samples that shared the same cell type (grouping), and genes with similar expression (hierarchical clustering, see Additional file 1, Figure S2). **A.** Pairwise: To control for environmental variation, samples grown in the same conditions were compared to identify differentially expressed genes (Additional file 9). **B.** Group: To increase statistical power we grouped samples of similar cell types (Additional file 10). **C**, **D**. Three methods, comparison of experimentally paired samples, comparison of groups of similar cell types, and hierarchical clustering, were used to identify with high confidence those genes upregulated in colonial cells (Additional file 11) (**C**) and thecate cells (Additional file 12) (**D**) as well as swimming cells (Additional file 13). See Fig. S1 for explanation of abbreviations.

**
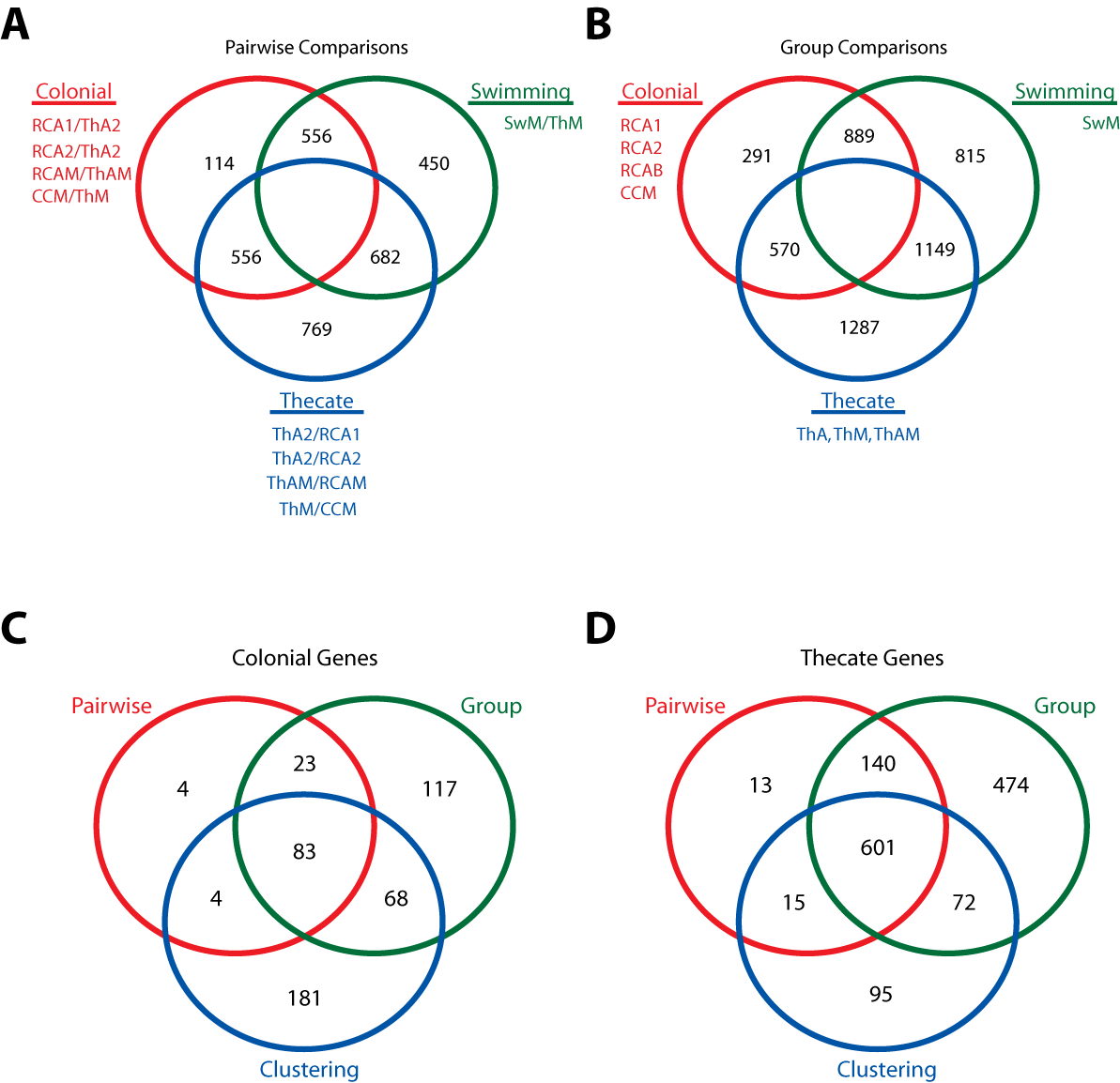
**

**Figure S4.**

**Gene expression correlates with cell type.** The expression pattern of genes identified as upregulated in either colonial (Additional file 11) or thecate (Additional file 12) cells in Additional file 1, Figure S3C and D are displayed as upregulated in yellow and downregulated in blue. Brackets (right) indicate paired samples from experiments 1 - 3. Similar cell types (left) have similar gene expression (see Fig. S1 for explanation of abbreviations). Values given at each node are the Pearson correlation coefficients for the least correlated pair of samples. Z-score reflects the number of standard deviations away from the mean.


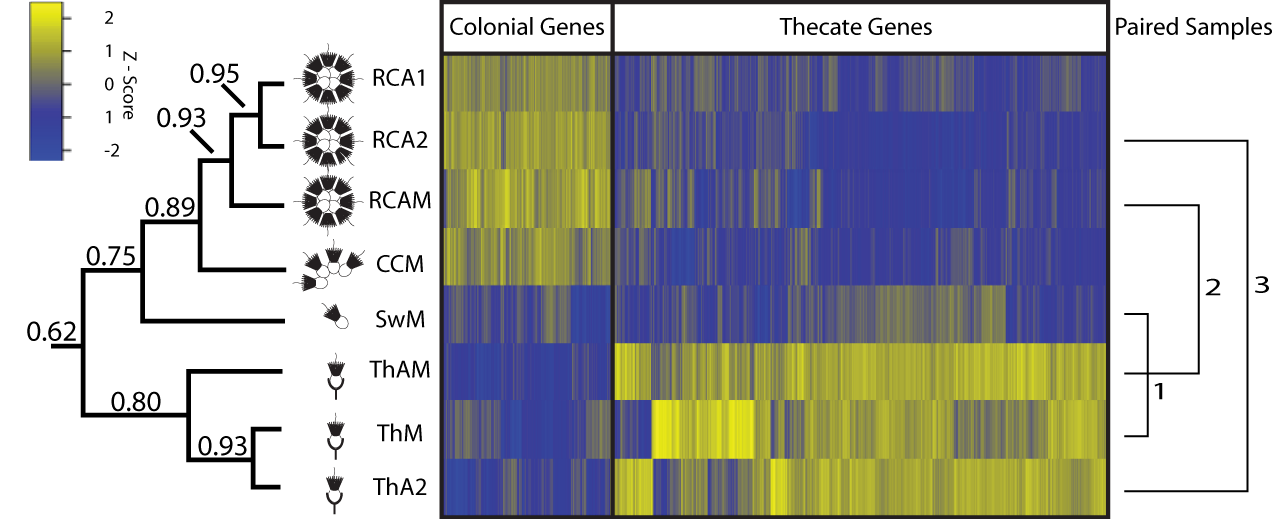


**Figure S5.**

***S. rosetta* cadherin expression.** Two of the 29 predicted cadherins encoded by the *S. rosetta* genome were upregulated in colonies relative to single cells, consistent with a role in colony biology. Additionally, six cadherins were specifically upregulated in thecate cells relative to colonies, consistent with a role in substrate attachment. The expression differences of the displayed genes in colonial cells vs. thecate cells have a Welch’s t-test p-value of less than 0.05.


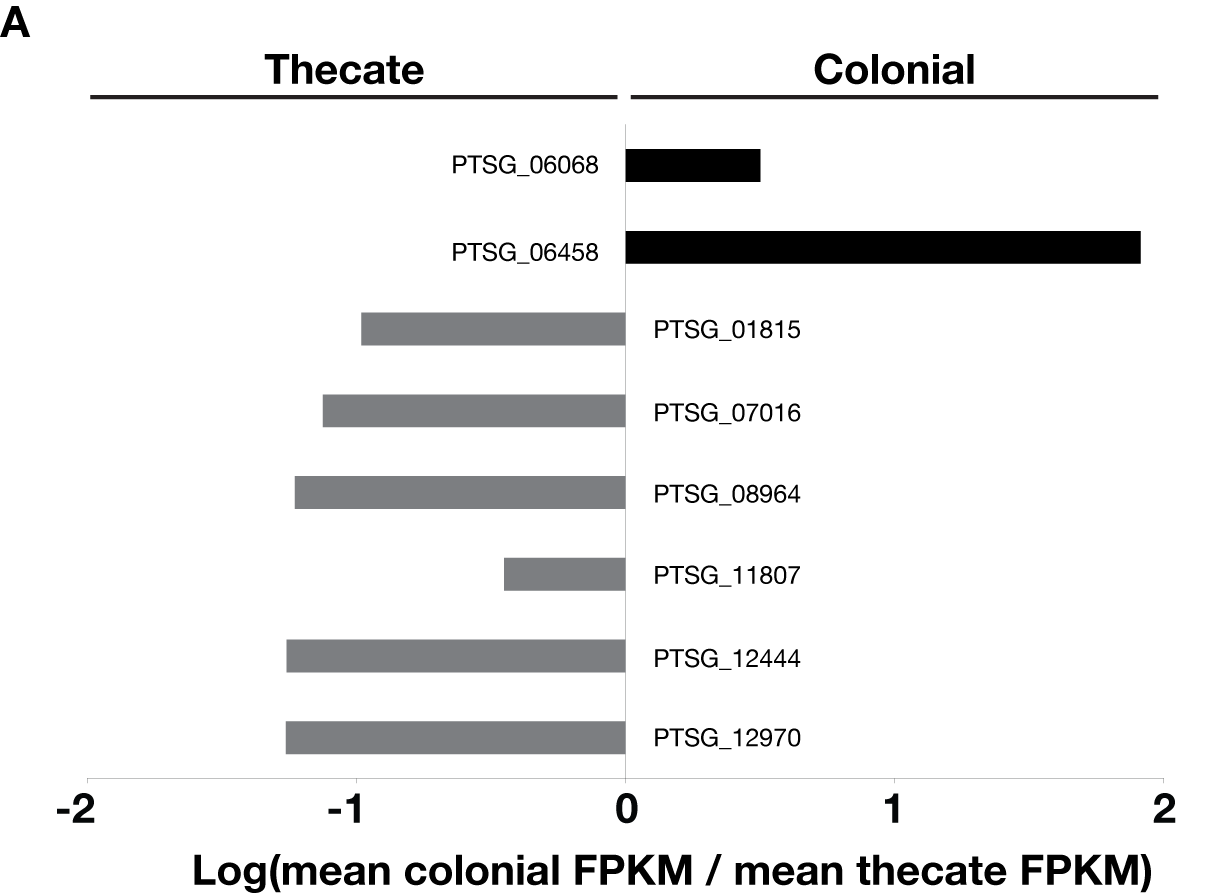


**Figure S6.**

**Hedgehog signal domain-encoding genes are upregulated in thecate and colonial cell types. A**. The *S. rosetta* genome encodes seven proteins predicted to contain a Hedghog signal domain (N-hh) that is always found N-terminal of a von Willebrand A domain (VWA). C-Lec, C-type Lectin; EC, Extracellular Cadherin; EGF, epidermal growth factor; Signal, signal peptide; TM, transmembrane; TNF, tumor necrosis factor. **B**. Genes encoding the Hedgehog signaling domain and a transmembrane domain were more highly expressed in thecate cells. Genes containing the Hedgehog signaling domain without a transmembrane domain were more highly expressed in colonial cells. X-axis is Log(mean colonial FPKM / mean attached FPKM). *Welch’s t-test p-value <0.05.


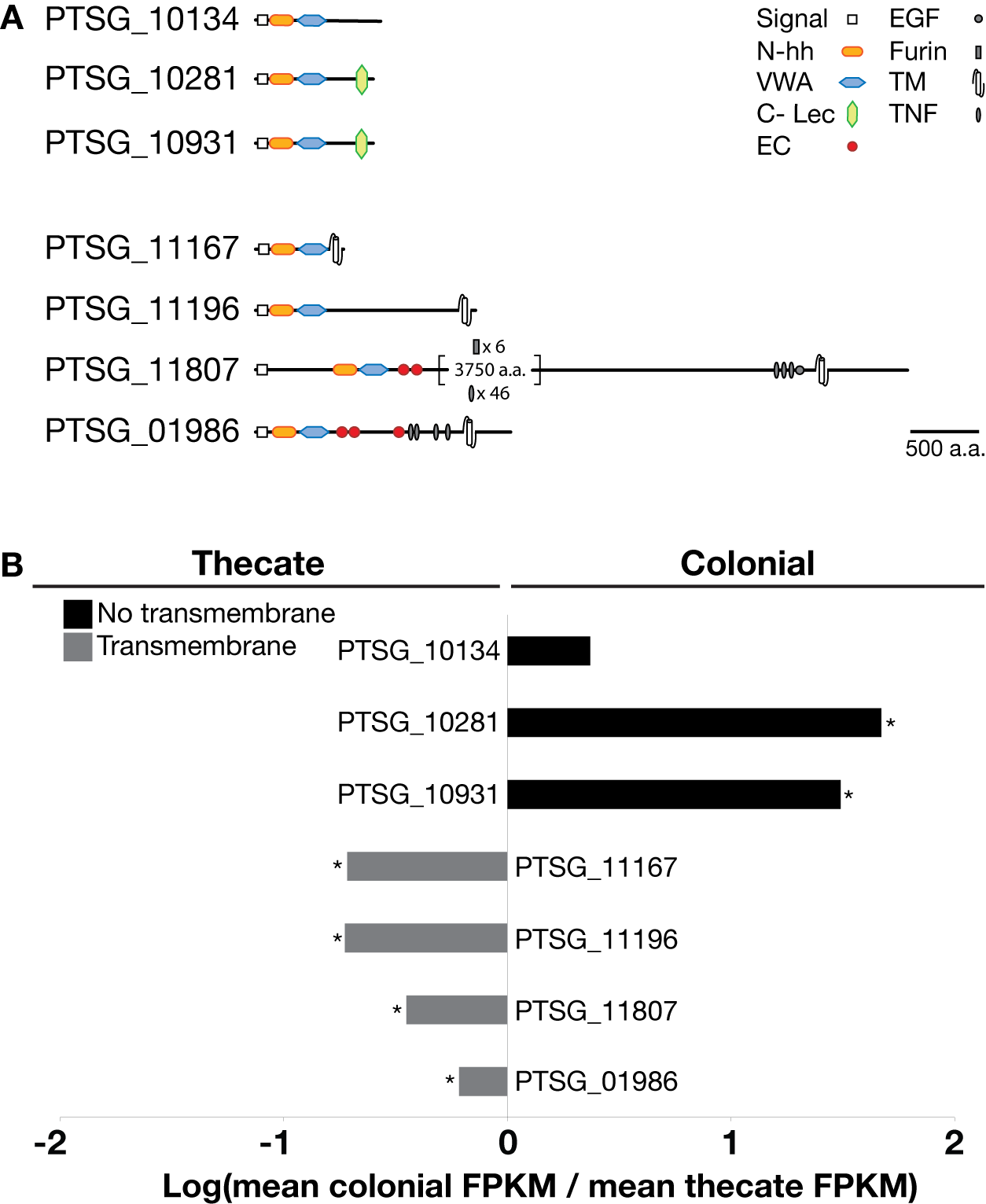


**Figure S7.**

**Protein domain architecture of *S. rosetta* septins.** Like most septins from metazoans and fungi , all four *S. rosetta* septins contain a conserved GTP_CDC binding domain with three conserved GTP binding motifs: G1, G3 and G4. The N-terminal polybasic region is shown in black and the Septin Unique Element is indicated in grey. A C-terminal coiled-coil domain is not detected in PTSG_04106. PTSG_04364* indicates the gene model revised based on sequencing of cDNA.


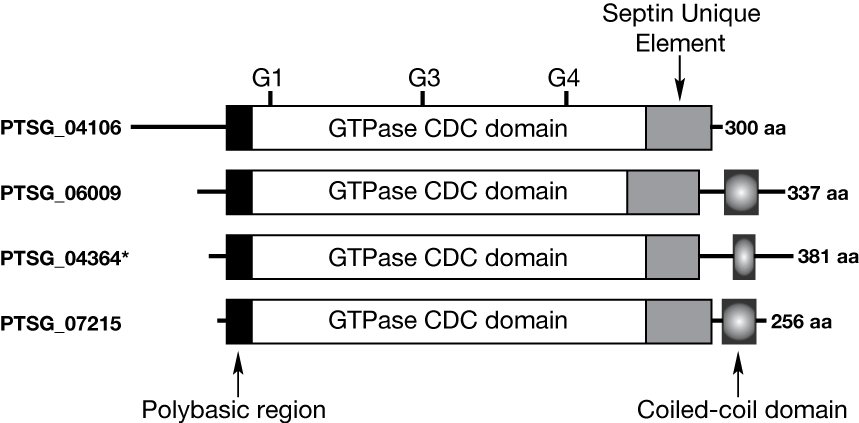


**Figure S8.**

**Septin sequence conservation.** The amino acid sequence alignment is shown for the four *S. rosetta* septins in addition to the *Drosophila melanogaster* and *Saccharomyces cerevisiae* septin homologs Pnut and Cdc3, respectively. Numbers at the end of each line indicate amino acid positions. Conservation across the alignment is shown with grey shading. The approximate position of the polybasic region is indicated in black at the N-terminus while predicted coiled-coil domains are underlined in purple at the C-terminus. The GTP_CDC binding domain is in the double-bound box with the conserved motifs G1 (GxxxxGK[ST]), G2 (DxxG), and G3 (xKxD) indicated above the corresponding amino acid sequence that is highlighted in red. Two additional residues that are highly conserved across septins are highlighted in the blue box directly following the G1 binding motif. The Septin Unique Element is bound by the black box. Residues that are conserved in at least 50% of the septin sequence examined by Momany, 2008 are indicated above the corresponding amino acid sequence. Bold typeface corresponds to residues that are conserved across at least 75% of these septins .


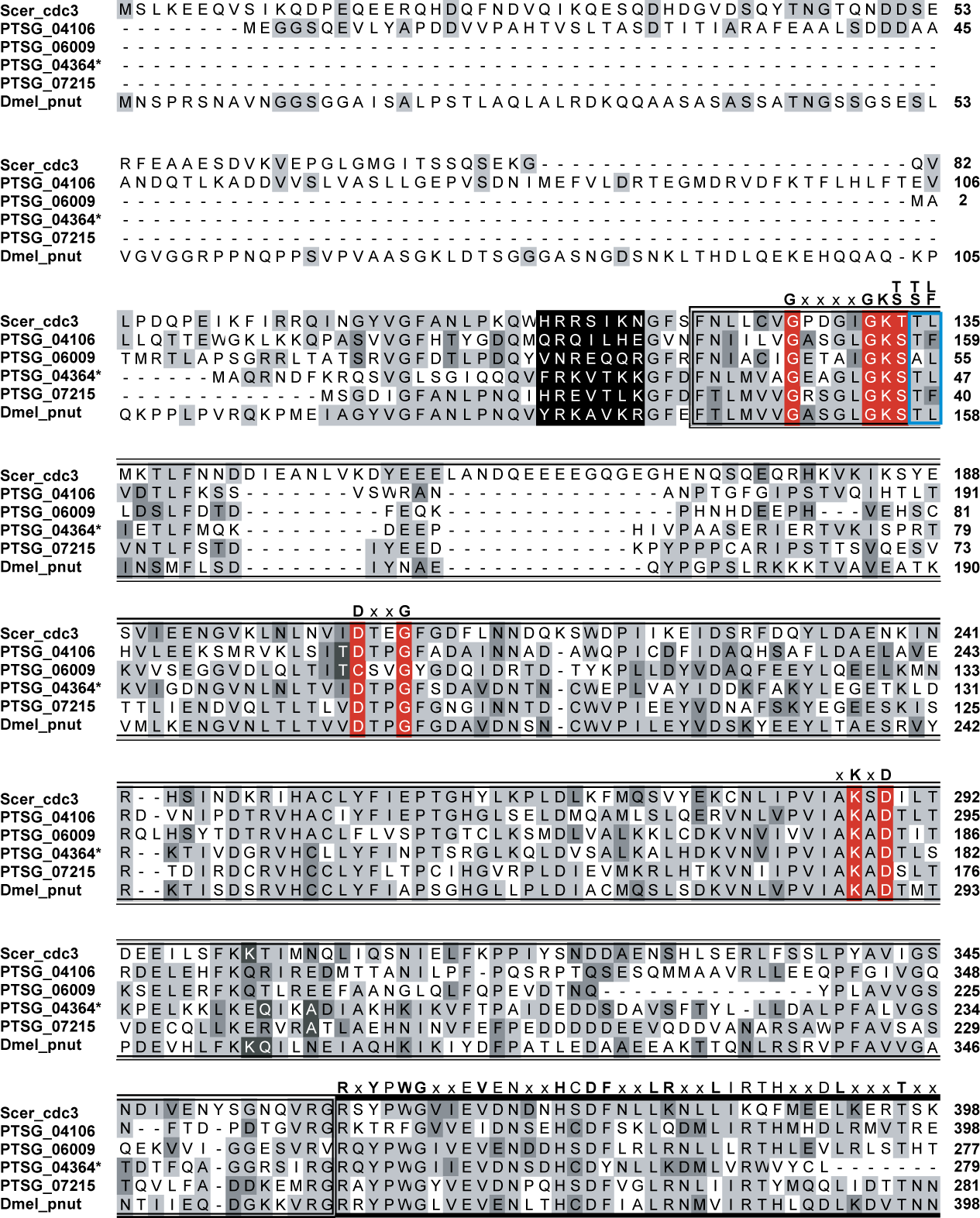

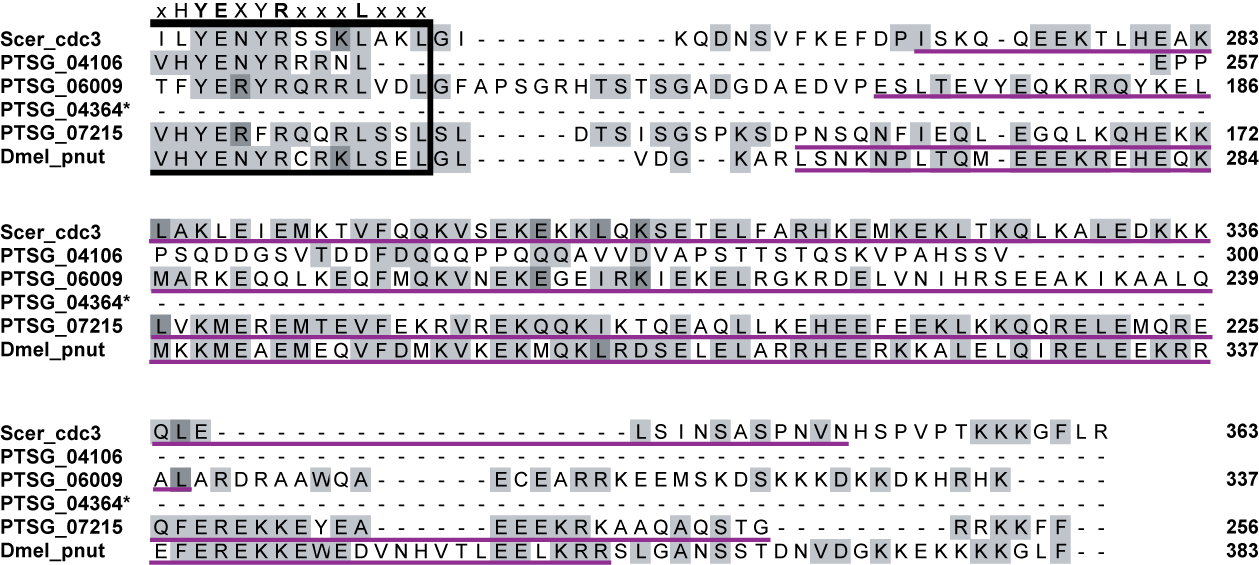


**Figure S9.**

**Septin gene family phylogeny.** The four *S. rosetta* amino acid sequences were added to a published septin data set from Momany *et al.* (2008) using the same gene identifiers. Maximum likelihood analysis of the explanded data set resulted in the shown topology, which includes many previously supported clades . There are two exceptions noted with *; the *Encephalitozoon cuniculi* septin 2 sequence (EcuSep2) clusters with Group 2A (CDC3) and SpoSpn 5 clusters with Group 3 (CDC11). The major groupings are indicated to the right of each corresponding clade and the four *S. rosetta* septins are highlighted in bold with red arrows. PTSG_04106 groups with Group 1A, PTSG_06009 nests within Group 1B, PTSG_04364* clusters with Group 2B, and PTSG_07215 nests within Group 4.


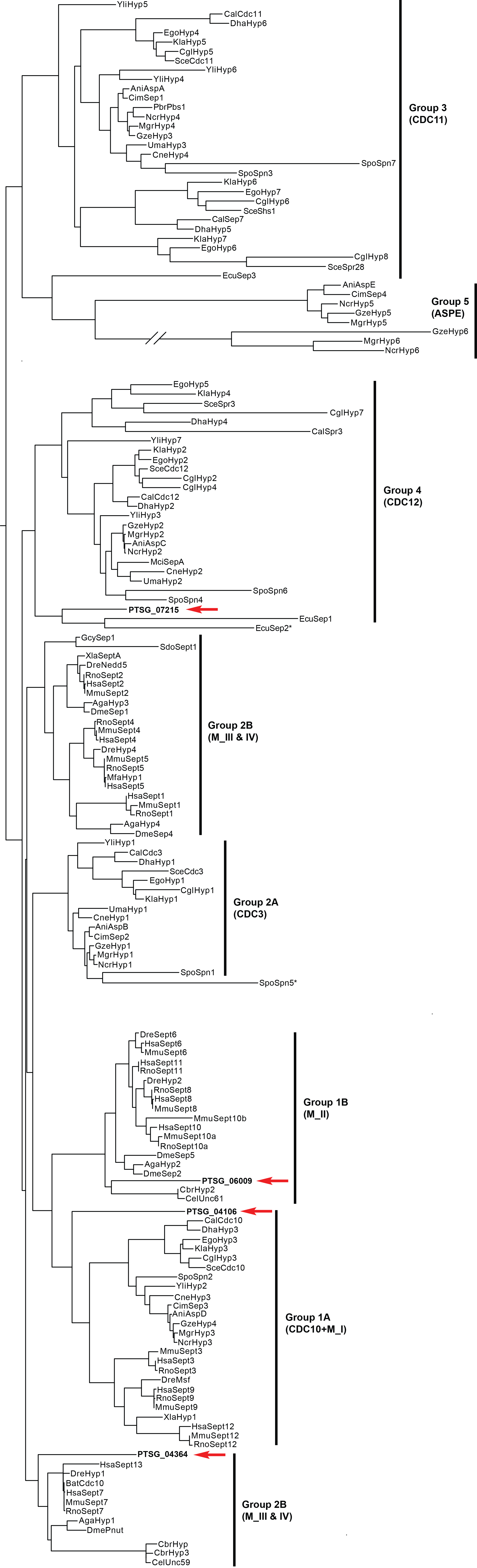


**Figure S10.**

**Ortholog cluster origin enrichment is robust to species composition**. To test the sensitivity of the ortholog cluster origin enrichment results (Fig. 4) to the species selected for analysis, we ran a jackknife analysis of 10,000 trials, each with a random subset of species, but always included *S. rosetta* and *M. brevicollis*. Results found to be statistically significant in the ortholog cluster origin enrichment analysis (Fig. 4) continued to deviate from expectation, while those not found to be significant do not deviate from expectation, regardless of species composition. Histogram color corresponds to ortholog cluster origin color used in Fig. 4 and Fig. S11 (red, *S. rosetta* unique; blue, choanoflagellate; yellow, choanimal; green, ancient).


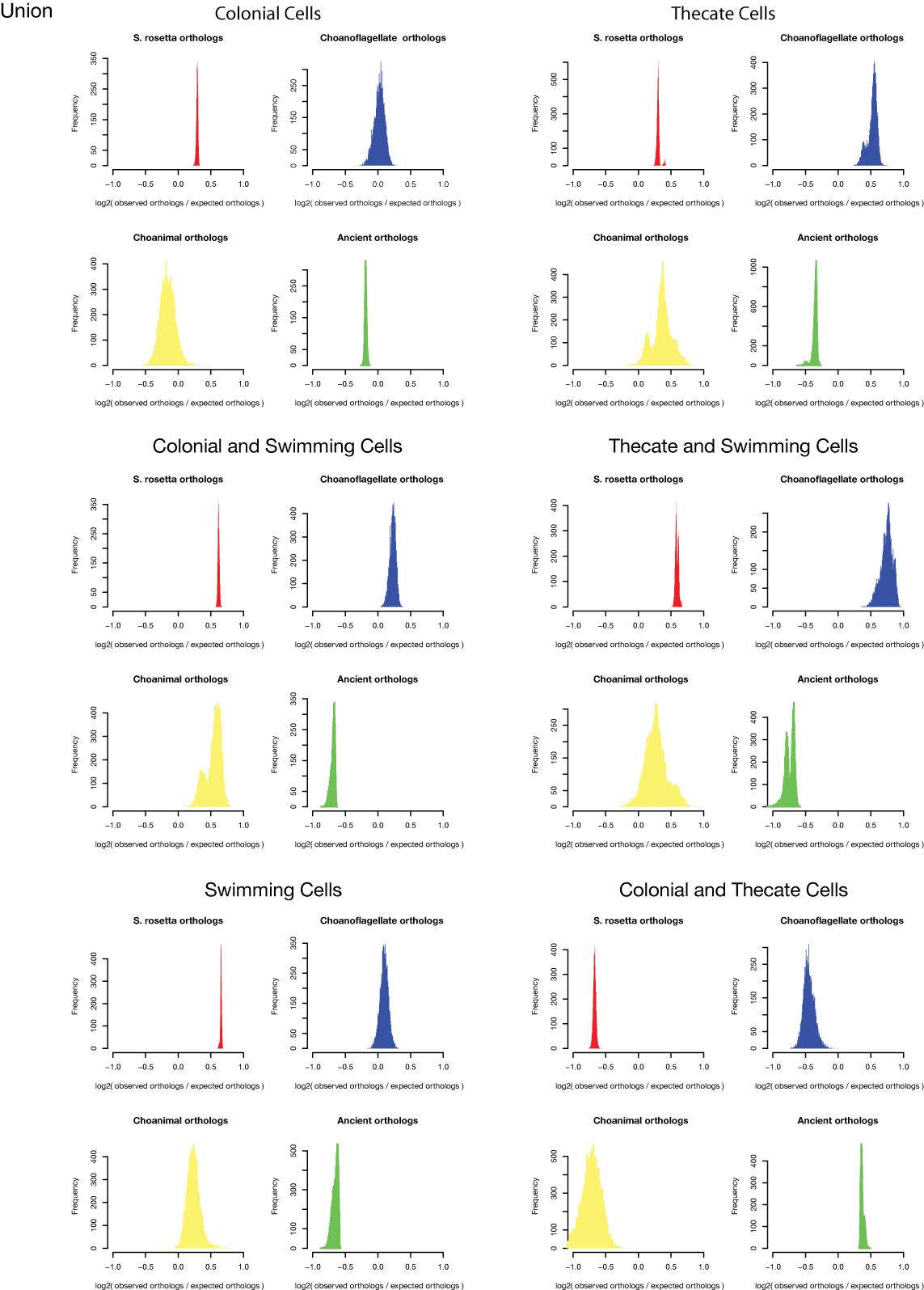


**Figure S11.**

**Ortholog cluster origin enrichment is robust to changes in MCL (Markov Cluster algorithm) species inflation value.** To test the sensitivity of phylogenic enrichment results to the Markov Clustering algorithm (MCL) inflation parameter, which controls inclusiveness of the ortholog clusters, we ran the MCL algorithm an additional 19 times with inflation values from 1.1 to 3. All 34 species were included (Table S4). The ratio of observed counts to expected counts remained relatively constant in all cases for non-extreme values, indicating that the enrichment analysis is robust.


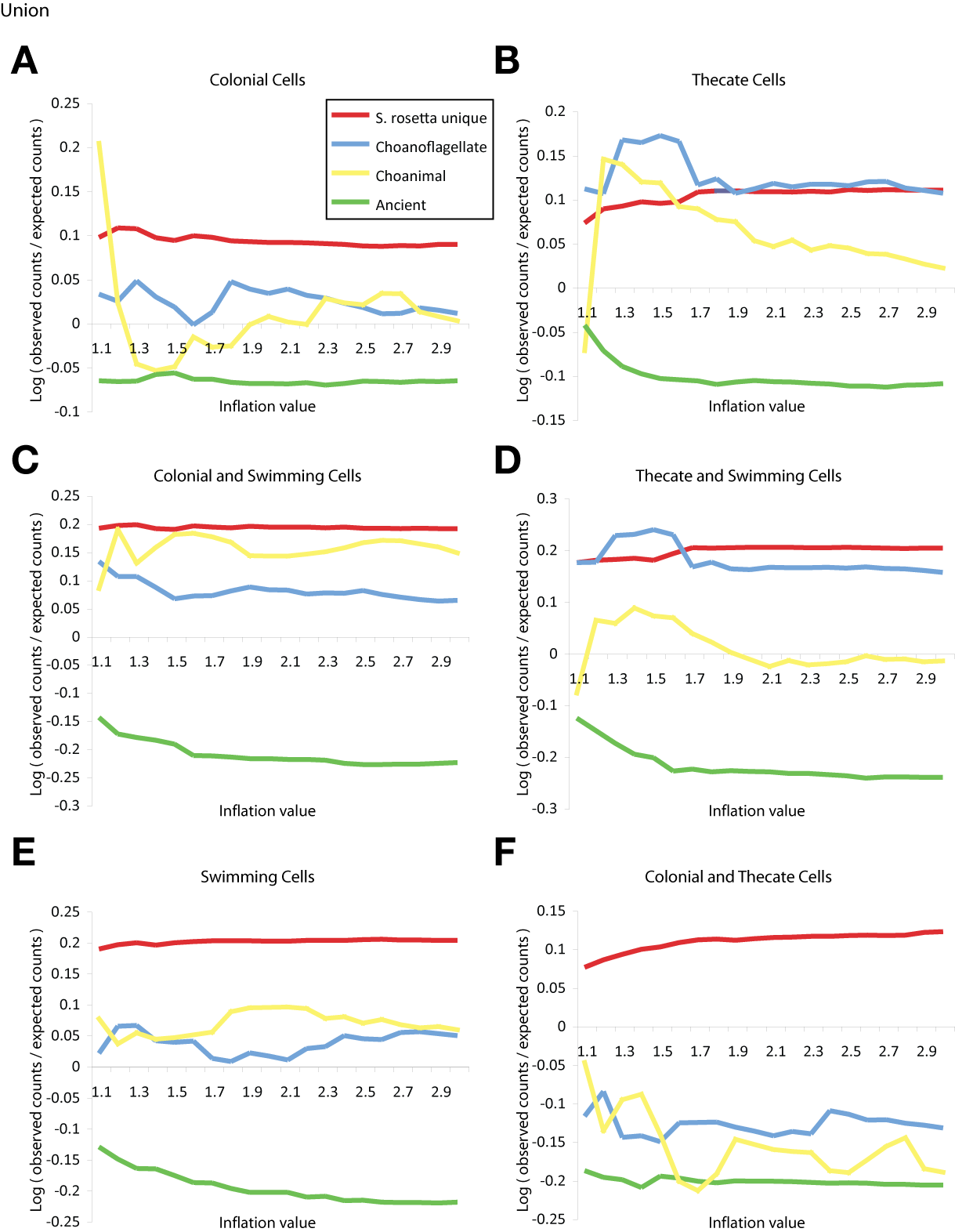


**Figure S12.**

**Expression levels of receptor tyrosine kinase families.** The tyrosine kinase families that are shared between *S. rosetta* and *M. brevicollis* (see Table S6) tended to be more highly expressed in the attached state, while those unique to *S. rosetta* are more highly expressed in the rosette state. Two TK families that are unique to *S. rosetta*, RTKO and RTKQ, are significantly differentially upregulated in rosette colonies (p < 0.05), similar to the whole-genome findings that genes unique to or expanded in *S. rosetta* are more highly expressed in the rosette state.


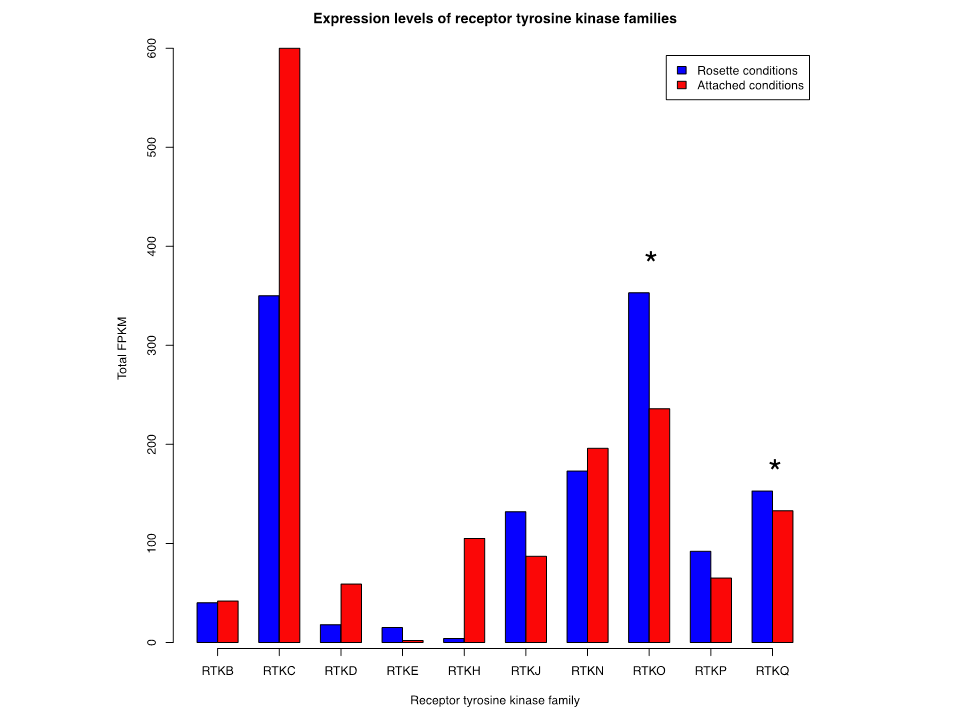


**Figure S13.**

**Metazoan developmental protein domains in choanoflagellates.** Despite a multicellular life stage being present in *S. rosetta* and thus far undetected in *M.* *brevicollis*, the developmental protein domain content between the two species is remarkably similar. The presence of a protein domain is indicated with a grey box. *S. rosetta* encodes a fibronectin type 2 domain that was not identified in *M. brevicollis*.


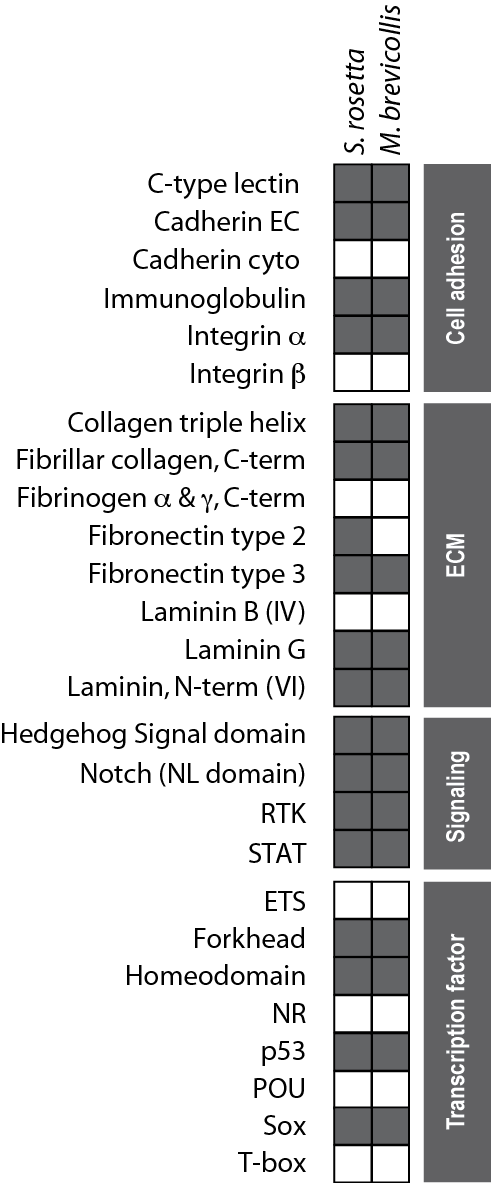


**Figure S14. Distribution of ortholog pairs detected in the *S. rosetta* and *M. brevicollis* genomes.** Vertical lines represent scaffolds from the *S. rosetta* and *M. brevicollis* genomes, arranged in order of predicted length. Grey lines connect orthologous loci shared between scaffolds from the *S. rosetta* (left) and *M. brevicollis* (right) genomes. No two scaffolds share more than 134 orthologs and all but three scaffolds have fewer than 100 ortholog pairs, revealing that there is relatively little conservation in physical linkage of ortholog pairs between *S. rosetta* and *M. brevicollis* (Additional file 2.xlsx).

***S. rosetta* *M. brevicollis***

**
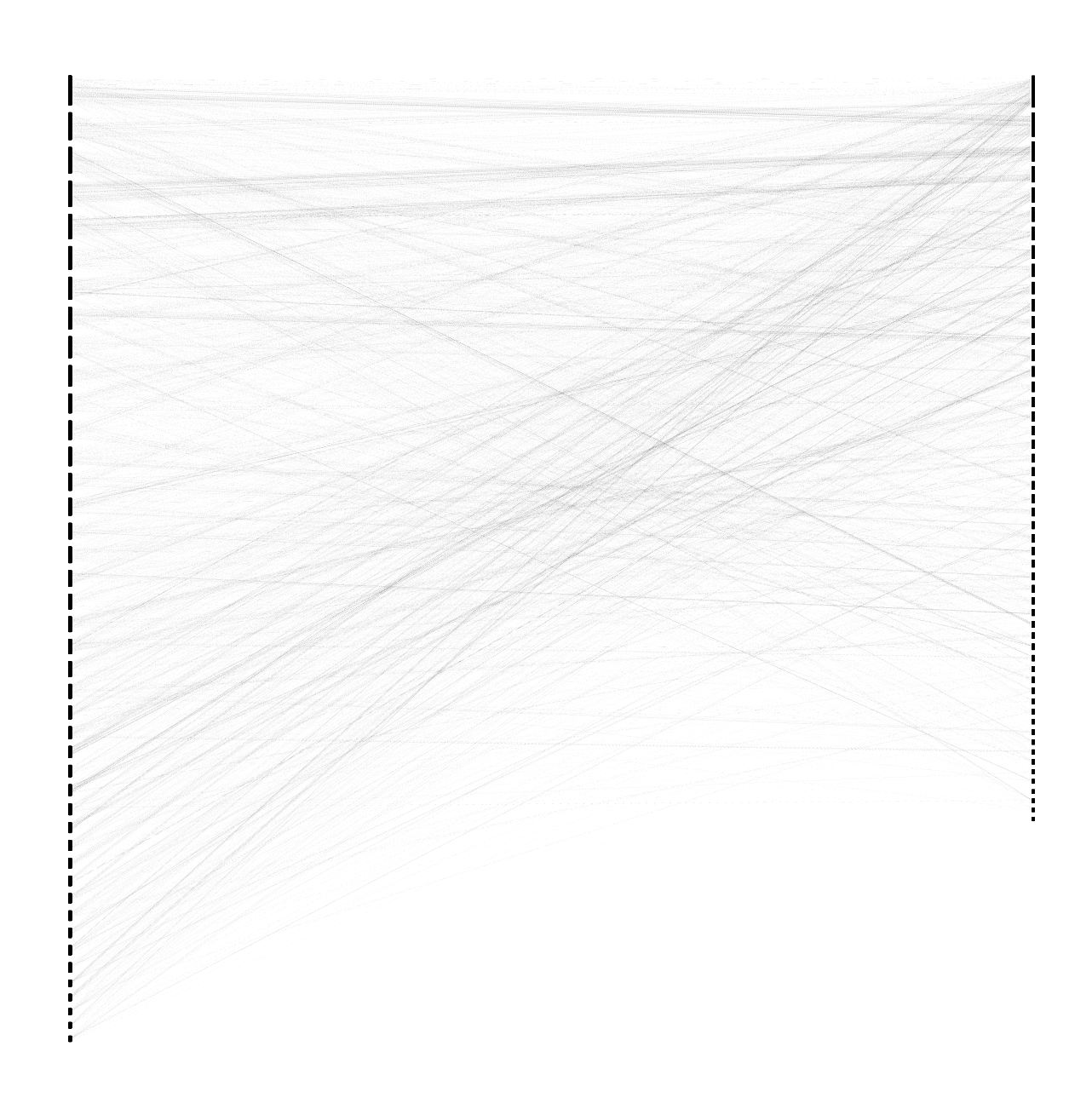
**

**Table S1. *S. rosetta* and *M. brevicollis* genome statistics.**

Category for Comparison *S. rosetta* *M. brevicollis*

Assembly Statistics

Number of Scaffolds 154 218

Scaffold N50 (Mb) 1.52 1.07

% Q40 98.51 N/A

Genome

Size (Mb) 55.44 41.63

Genome coverage 33.07x 8.39x

% GC Content 56.01 % 54.89%

Genes

Number of genes 11,629 9,171

Median gene length 3,220 2,183

% Genes with transcript support 98% 46%

Exons

Number of exons 100,147 69,682

Median exon length 117 124

Introns

Intergenic 10,312 9,282

Number of introns 88,594 60,636

Mean intron length (bp) 255 174

Mean introns per gene 7.5 6.6

**Table S2. Mapping of *de novo* transcript assembly.**

| Library name | Library id number | Number of transcripts | Number mapped | Percent mapped |
| --- | --- | --- | --- | --- |
| RCA1 | 33719 | 11925 | 11450 | 96.02 |
| CCB | 33720 | 10093 | 9677 | 95.88 |
| RCA2 | 33721 | 12925 | 12438 | 96.23 |
| ThA2 | 33722 | 11791 | 11374 | 96.46 |
| RCAB | 33723 | 13951 | 13440 | 96.34 |
| ThAB | 33724 | 10354 | 10005 | 96.63 |
| SwB | 33725 | 13377 | 12897 | 96.41 |
| ThB | 33726 | 11050 | 10646 | 96.34 |

**Table S3. Telomeres predicted in the *S. rosetta* genome.**

**Super- Size End TTAGGG 40 kb Missing Matches**

**contig (kbp) Assembled Mates kb others**

1 2,682 5’ No 1 40 No

2 2,480 5’ No 4 10 Yes

2 2,480 3’ No 3 Yes

3 2,356 5’ Yes - 0 Yes

3 2,356 3’ No - - Yes

4 2,372 3’ No - - Yes

5 2,237 5’ No - - Yes

5 2,237 3’ No - - Yes

6 2,078 3’ No 4 10 Yes

7 2,024 5’ No 4 30 Yes

7 2,024 3’ No - - Yes

8 1,951 3’ Yes 13 0 Yes

9 1,890 5’ No 9 15 Yes

9 1,890 3’ No - - Yes

10 1,852 5’ No - - Yes

10 1,852 3’ No 8 5 Yes

11 1,685 3’ No - - Yes

12 1,669 5’ No 1 35 No

12 1,669 3’ No 9 15 Yes

13 1,638 3’ No 16 10 Yes

14 1,475 5’ No - - Yes

14 1,475 3’ No - - Yes

15 1,520 5’ No - - Yes

15 1,520 3’ No 11 5 Yes

16 1,370 3’ No 3 15 Yes

17 1,374 5’ No 7 10 Yes

17 1,374 3’ No - - Yes

18 1,385 5’ No 1 5 Yes

18 1,385 3’ Yes 5 0 Yes

19 1,260 5’ No 3 30 Yes

20 1,278 5’ No - - Yes

20 1,278 3’ No - - Yes

21 1,216 5’ No 2 5 Yes

22 1,286 5’ No 3 10 Yes

22 1,286 3’ No 3 10 Yes

23 1,192 5’ Yes 9 0 Yes

24 1,067 5’ No 2 10 Yes

24 1,067 3’ No 10 15 Yes

25 983 5’ No 2 5 Yes

26 897 3’ No 1 5 Yes

27 927 5’ No 1 30 Yes

**Super- Size End TTAGGG 40 kb Missing Matches**

**contig (kbp) Assembled Mates kb others**

27 927 3’ No - - Yes

28 911 5’ No 1 10 Yes

29 916 5’ Yes 7 0 Yes

29 916 3’ No 6 10 Yes

30 789 5’ No - - Yes

31 796 5’ No 3 15 Yes

31 796 3’ No 3 10 Yes

32 786 5’ No - - Yes

32 786 3’ No 1 20 Yes

33 740 5’ No 4 15 Yes

33 740 3’ No 1 35 Yes

34 739 5’ No 1 10 Yes

34 739 3’ No 1 35 No

35 753 5’ No 10 5 Yes

36 741 5’ No 2 30 Yes

37 725 5’ Yes - - Yes

37 725 3’ No - - Yes

38 740 3’ No - - Yes

39 427 5’ No - - Yes

40 447 3’ No 2 5 Yes

41 388 3’ No - - Yes

42 358 3’ No 2 35 Yes

43 321 5’ No - - Yes

44 82 5’ No - - Yes

45 36 3’ No 9 5 No

**Table S4. Genomes used for comparative genomics**


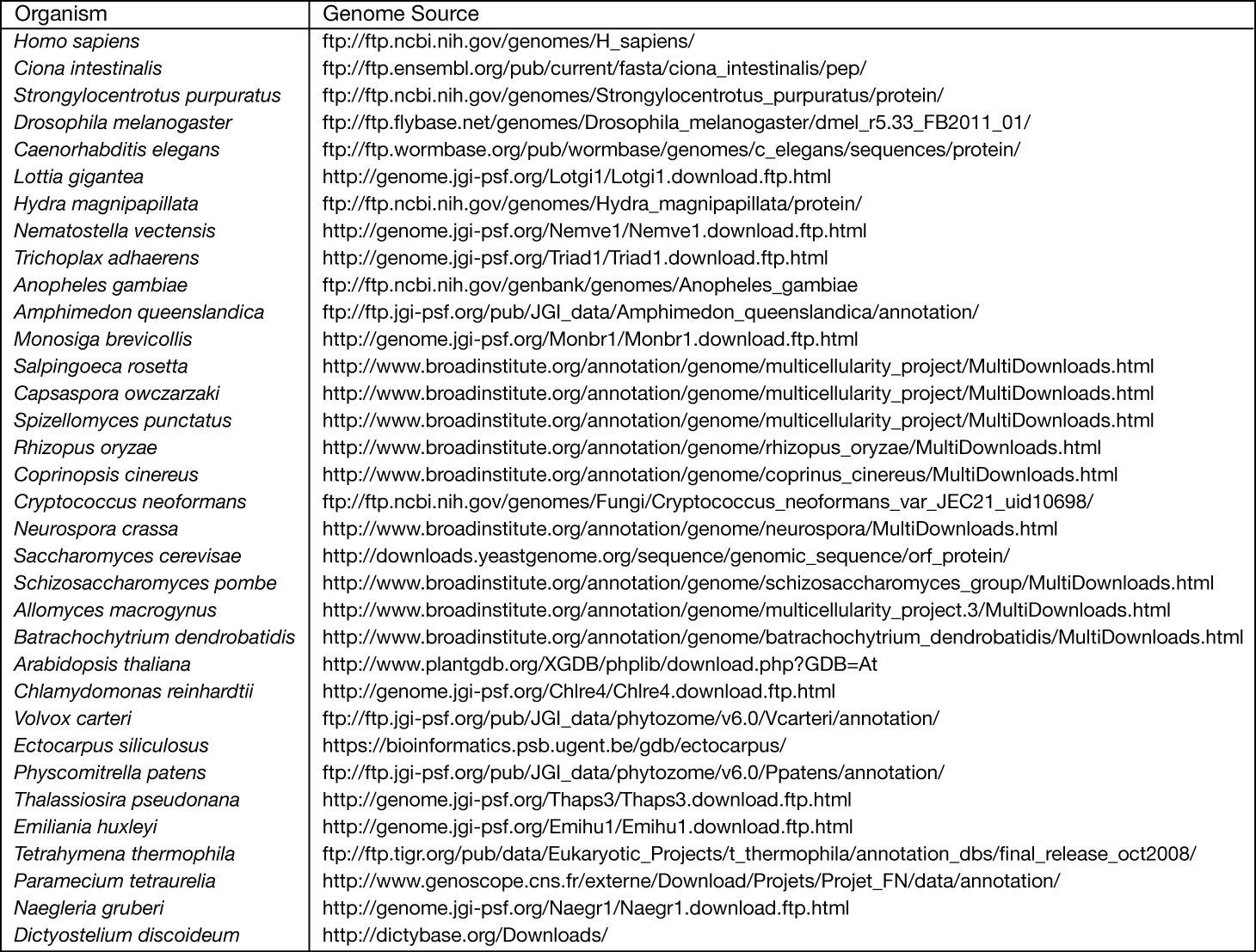


**Table S5. Gene Ontology enrichment of novel genes.** Ortholog clusters identified as novel gains for a given organism or reconstructed ancestor were annotated using Gene Ontology terms and tested for enrichment against the organismal or reconstructed ancestral genome using Ontologizer 2.0.

**
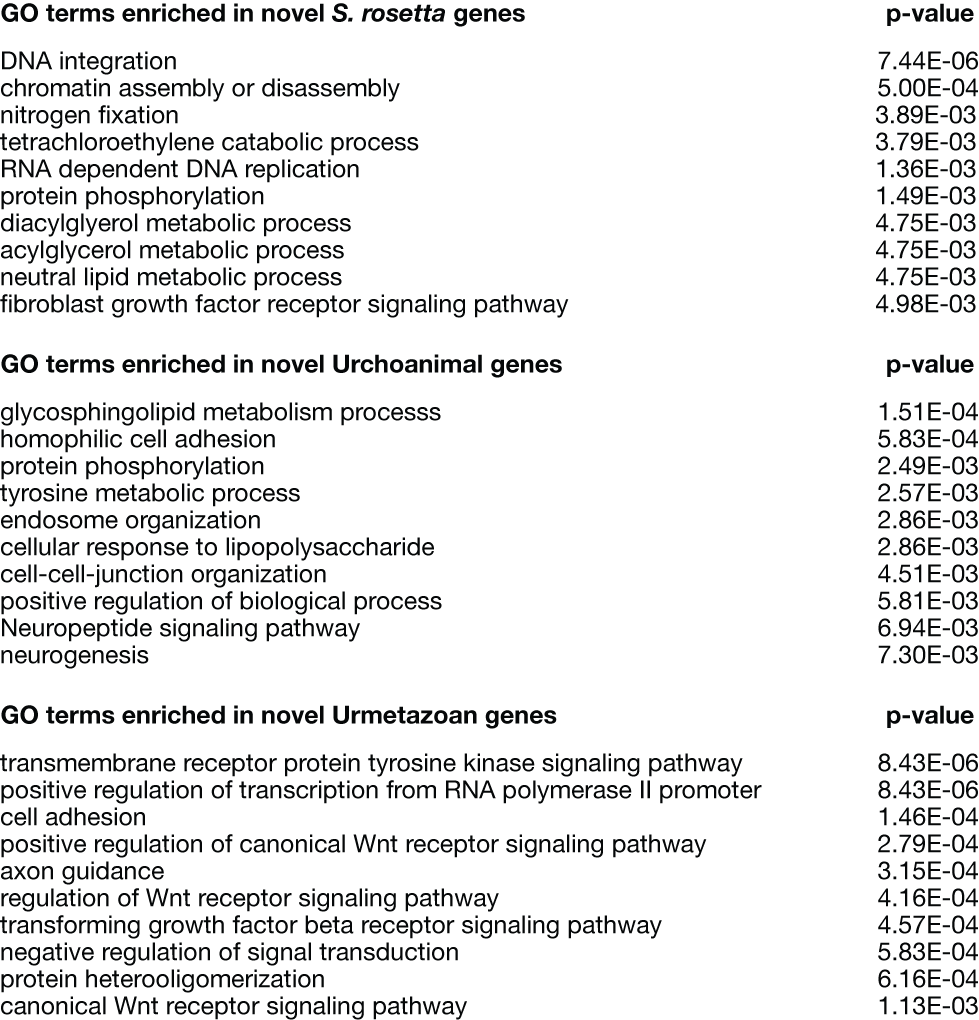
**

**Table S6. *S. rosetta* tyrosine kinases.**

| **Family** | **In Metazoa?** | **Receptor?** | ***S. rosetta*** | ***M. brevicollis*** | **In Common** |
| --- | --- | --- | --- | --- | --- |
| Abl | **Y** | **N** | 2 | 2 | 2 |
| Csk | **Y** | **N** | 1 | 1 | 1 |
| FAK | **Y** | **N** | 1 | 0 | 0 |
| Fer | **Y** | **N** | 1 | 0 | 0 |
| Src | **Y** | **N** | 2 | 4 | 2 |
| Syk/Shark | **Y** | **N** | 1 | 1 | 1 |
| Tec | **Y** | **N** | 1 | 1 | 1 |
| Eph | **Y** | Y | 1 | 2 | 1 |
| RTKS | (InsR)* | Y | 5 | 3 | 3 |
| CTKA | N | **N** | 1 | 2 | 1 |
| CTKB | N | **N** | 2 | 2 | 2 |
| FYTK | N | **N** | 1 | 2 | 1 |
| HMTK | N | **N** | 15 | 15 | 15 |
| FGTK | N | Y | 0 | 14 | 0 |
| LRTK | N | Y | 0 | 5 | 0 |
| RTKA | N | Y | 0 | 9 | 0 |
| RTKB | N | Y | 1 | 9 | 1 |
| RTKC | N | Y | 9 | 9 | 1 |
| RTKD | N | Y | 1 | 4 | 1 |
| RTKE | N | Y | 1 | 6 | 1 |
| RTKF | N | Y | 0 | 3 | 0 |
| RTKG | N | Y | 0 | 2 | 0 |
| RTKH | N | Y | 1 | 2 | 1 |
| RTKJ | N | Y | 2 | 2 | 1 |
| RTKK | N | Y | 0 | 2 | 0 |
| RTKL | N | Y | 0 | 4 | 0 |
| RTKM | N | Y | 0 | 2 | 0 |
| RTKN | N | Y | 7 | 2 | 1 |
| RTKO | N | Y | 3 | 0 | 0 |
| RTKP | N | Y | 3 | 0 | 0 |
| RTKQ | N | Y | 2 | 1 | 1 |
| RTKR | N | Y | 4 | 3 | 3 |
| RTKT | N | Y | 1 | 1 | 1 |
| TK-Unique | N | Uncertain** | 12 | 11 | 0 |
| UTKA | N | Uncertain** | 1 | 1 | 1 |
| UTKB | N | Uncertain** | 3 | 1 | 1 |
| UTKC | N | Uncertain** | 1 | 1 | 1 |
| UTKD | N | Uncertain** | 2 | 2 | 2 |
| UTKE | N | Uncertain** | 1 | 1 | 1 |
| UTKF | N | Uncertain** | 1 | 1 | 1 |
| UTKG | N | Uncertain** | 1 | 1 | 1 |
| UTKH | N | Uncertain** | 1 | 1 | 1 |
|  |  |  |  |  |  |
| **Total** |  |  | **92** | **135** | **51** |

*****RTKS genes are very similar to InsR, but not definitively so, so we kept a unique family name (RTKS).

**Diagnostic features of typical receptor TKs (e.g. sigP or TM domain) and typical cytoplasmic TKs (e.g. SH2, SH3, PH, and C2 domains) were not detected.

**Table S7. Phylogenetic distribution of genes upregulated in different cell types.** Ortholog clusters were annotated as ancient, choanimal, choanoflagellate or *S. rosetta*-unique based on the cluster member detected in the species most distantly related to *S. rosetta* and the proportion of annotations calculated for the entire *S. rosetta* genome (Figure 4A). Genes upregulated in different cell types were tested for phylogenic enrichment by comparing their annotation counts to frequencies for the entire genome and are displayed graphically in Figure 4B-E.

|  | Genes Upregulated In Thecate Cells | | | | |
| --- | --- | --- | --- | --- | --- |
|  | Observed Gene Count | Percent of Total | Expected Gene Count | Percent Enrichment | p-value |
| *S. rosetta* | 551 | 39% | 444 | 24% | 1.65 E-09 |
| Choano. | 134 | 10% | 91 | 48% | 7.09 E-06 |
| Choanimal | 95 | 7% | 73 | 31% | 8.11 E-03 |
| Ancient | 630 | 45% | 802 | -21% | 3.98 E-20 |
|  |  |  |  |  |  |
|  | Genes Upregulated In Colonial Cells | | | | |
|  | Observed Gene Count | Percent of Total | Expected Gene Count | Percent Enrichment | p-value |
| *S. rosetta* | 187 | 39% | 151 | 24% | 4.68 E-04 |
| Choano. | 32 | 7% | 31 | 4% | 7.37 E-01 |
| Choanimal | 22 | 5% | 25 | -11% | 6.62 E-01 |
| Ancient | 239 | 50% | 273 | -13% | 2.00 E-03 |
|  |  |  |  |  |  |
|  | Genes Upregulated In Colonial and Swimming Cells | | | | |
|  | Observed Gene Count | Percent of Total | Expected Gene Count | Percent Enrichment | p-value |
| *S. rosetta* | 443 | 48% | 288 | 54% | <1.0 E-50 |
| Choano. | 68 | 7% | 59 | 16% | 1.90 E-01 |
| Choanimal | 71 | 8% | 47 | 51% | 6.17 E-04 |
| Ancient | 333 | 36% | 521 | -36% | 2.39 E-35 |
|  |  |  |  |  |  |
|  | Genes Upregulated In Swimming Cells | | | | |
|  | Observed Gene Count | Percent of Total | Expected Gene Count | Percent Enrichment | p-value |
| *S. rosetta* | 426 | 50% | 271 | 57% | <1.0 E-50 |
| Choano. | 60 | 7% | 55 | 9% | 4.51 E-01 |
| Choanimal | 49 | 6% | 44 | 11% | 4.15 E-01 |
| Ancient | 324 | 38% | 489 | -34% | 2.00 E-29 |

**Table S8.**

**Manual curation of gene families predicted to be absent from choanoflagellates by OrthoMCL.**

|  | ***S. rosetta*** | | ***M. brevicollis*** | |
| --- | --- | --- | --- | --- |
| **Family** | **Manual** | **OrthoMCL** | **Manual** | **OrthoMCL** |
| Tetraspanin | 1 | No | 1 | No |
| delta-cantenin | No | No | No | No |
| beta-laminin | No | No | No | No |
| TGFB | No | No | No | No |
| TGFB-receptor | No | No | No | No |
| Smad | No | No | No | No |
| WNT | No | No | No | No |
| WNTless | No | No | No | No |
| b-catenin | No | No | No | No |
| TCF/LEF | No | No | No | No |

References:

1. Momany M, Pan F, Malmberg RL: **Evolution and Conserved Domains of the Septins.** In *The Septins.* John Wiley & Sons, Ltd; 2008: 35-45

2. Versele M, Thorner J: **Septin collar formation in budding yeast requires GTP binding and direct phosphorylation by the PAK, Cla4.** *The Journal of cell biology* 2004, **164:**701-715.
